# Complexin has a dual synaptic function as checkpoint protein in vesicle priming and as a promoter of vesicle fusion

**DOI:** 10.1073/pnas.2320505121

**Published:** 2024-04-03

**Authors:** Francisco José López-Murcia, Kun-Han Lin, Manon M. M. Berns, Mrinalini Ranjan, Noa Lipstein, Erwin Neher, Nils Brose, Kerstin Reim, Holger Taschenberger

**Affiliations:** ^a^Department of Molecular Neurobiology, Max Planck Institute for Multidisciplinary Sciences, Göttingen 37075, Germany; ^b^Laboratory of Membrane Biophysics, Max Planck Institute for Multidisciplinary Sciences, Göttingen 37077, Germany; ^c^Göttingen Graduate School for Neurosciences, Biophysics, and Molecular Biosciences, Georg August University Göttingen, Göttingen 37077, Germany; ^d^Cluster of Excellence ‘Multiscale Bioimaging’, Georg August University Göttingen, Göttingen 37073, Germany

**Keywords:** synaptic transmission, short-term plasticity, synaptic vesicle priming, calyx of Held, numerical simulation

## Abstract

The presynaptic SNARE-complex regulator complexin (Cplx) enhances the fusogenicity of primed synaptic vesicles (SVs). Consequently, Cplx deletion impairs action potential-evoked transmitter release. Conversely, though, Cplx loss enhances spontaneous and delayed asynchronous release at certain synapse types. Using electrophysiology and kinetic modeling, we show that such seemingly contradictory transmitter release phenotypes seen upon Cplx deletion can be explained by an additional of Cplx in the control of SV priming, where its ablation facilitates the generation of a “faulty” SV fusion apparatus. Supporting this notion, a sequential two-step priming scheme, featuring reduced vesicle fusogenicity and increased transition rates into the faulty primed state, reproduces all aberrations of transmitter release modes and short-term synaptic plasticity seen upon Cplx loss. Accordingly, we propose a dual presynaptic function for the SNARE-complex interactor Cplx, one as a “checkpoint” protein that guarantees the proper assembly of the fusion machinery during vesicle priming, and one in boosting vesicle fusogenicity.

## Significance

The SNARE-complex regulator complexin (Cplx) is expressed at virtually all chemical synapses. The role of Cplx as a positive regulator of evoked transmitter release is undisputed, but its function in spontaneous release is controversially discussed. We analyzed deficits in spontaneous, synchronous, and delayed asynchronous transmitter release at mammalian brain synapses following genetic Cplx ablation and developed and validated a kinetic scheme accurately describing synaptic vesicle (SV) priming and fusion. Based on this, we assign an additional function to Cplx, that of a “checkpoint” protein during SV fusion machinery assembly. The checkpoint model reconciles conflicting observations regarding the alleged functions of Cplx in transmitter release and challenges the view that Cplx is a SV “fusion clamp.”

Complexins (Cplxs) are essential regulators of soluble N-ethylmaleimide-sensitive factor attachment protein receptor (SNARE) complex function in neurotransmitter release at chemical synapses (1, 2). Their genetic deletion causes a reduction of action potential (AP)-evoked transmitter release, presumably reflecting a lower fusion-propensity of synaptic vesicles (SVs) docked at presynaptic release sites (3–7). However, it can also exert contradictory effects on spontaneous release at rest and delayed asynchronous release following conditioning stimulation in certain synapse types (6, 8). Synapse numbers and the pool of readily releasable SVs are mostly unaffected by Cplx loss (3, 4, 6, 9). When interpreting Cplx deletion-induced changes in synaptic strength and short-term plasticity (STP), it is important to realize that docked and primed SVs are functionally heterogeneous and that the dynamic regulation of SV priming states can account for variable transmission strength and diverse STP patterns (10–18). Considering that docked and primed SVs may reside in distinct priming states, of which only one is molecularly prepared for fusion (“fusion-competent”), and assuming fast and reversible state transitions, synaptic strength depends on two probabilities: i) the probability of a fusion-competent SV to fuse after AP arrival and ii) the probability that a given release site is occupied by a fusion-competent SV. These probabilities are difficult to distinguish experimentally and to assign to Cplx function.

Conspicuously enhanced delayed release was observed upon Cplx loss at certain mammalian glutamatergic CNS synapses after stimulation with single APs (19) or AP trains (6, 8). This is difficult to reconcile with the reduced SV fusogenicity after Cplx loss, stressing that the current understanding of Cplx function in transmitter release is inadequate. Delayed or asynchronous release, which is widespread in the CNS (20, 21), is triggered by residual presynaptic [Ca^2+^]_i_ which can remain elevated for up to hundreds of milliseconds after presynaptic AP firing (22–24). Residual [Ca^2+^]_i_ typically ranges from hundreds of nM to a few µM and potentially triggers asynchronous release by activating high-affinity Ca^2+^ sensor proteins that differ from those triggering synchronous release. Synaptotagmin 7 (Syt7) is a high-affinity Ca^2+^ sensor expressed at some presynaptic terminals of the mammalian CNS (25–28) but whether it is linked to aberrant delayed release observed after genetic Cplx loss is unknown.

The present study was designed to mechanistically define the differential roles of Cplx in the key transmitter release modes. Specifically, we wanted to determine i) whether Cplx increases the probability of SVs to fuse after AP arrival (*p_fusion_*) or the probability of primed SVs to be in the fusion-competent state, ii) whether aberrant delayed release is a general feature of mammalian Cplx-deficient synapses or restricted to a specific subset, iii) whether aberrant delayed release in Cplx-deficient synapses involves the high-affinity Ca^2+^ sensor Syt7, and, iv) whether aberrant delayed release upon Cplx loss results from a previously overlooked defect in SV priming.

To this end, we combined electrophysiology and mathematical modeling to characterize three mammalian synapses that only contain the Cplx1 paralog [glutamatergic calyx of Held synapses, glycinergic synapses between principal neurons (PNs) of the medial nucleus of the trapezoid body and their target neurons in the lateral superior olive (MNTB→LSO) of the auditory brainstem, and GABAergic synapses between molecular layer interneurons of the cerebellar cortex and Purkinje cells (MLI→PC)] upon genetic ablation of Cplx1 alone or Cplx1 and Syt7 together. Our data show that the reduced strengths of Cplx-deficient synapses are primarily due to reduced *p_fusion_*. Supported by numerical simulations reproducing delayed release at control and mutant synapses, we propose that Cplx has an additional role as a “checkpoint” factor in SV priming, so that in its absence an SV priming defect arises where SVs can transiently associate with a “faulty” fusion apparatus, leading to an altered Ca^2+^ dependence for vesicle fusion that fully accounts for the complex timecourse of aberrant delayed release after AP trains.

## Results

### Cplx Controls Synaptic Strength by Regulating the Fusion Probability of Tightly Docked SVs.

**Fig. 1.**
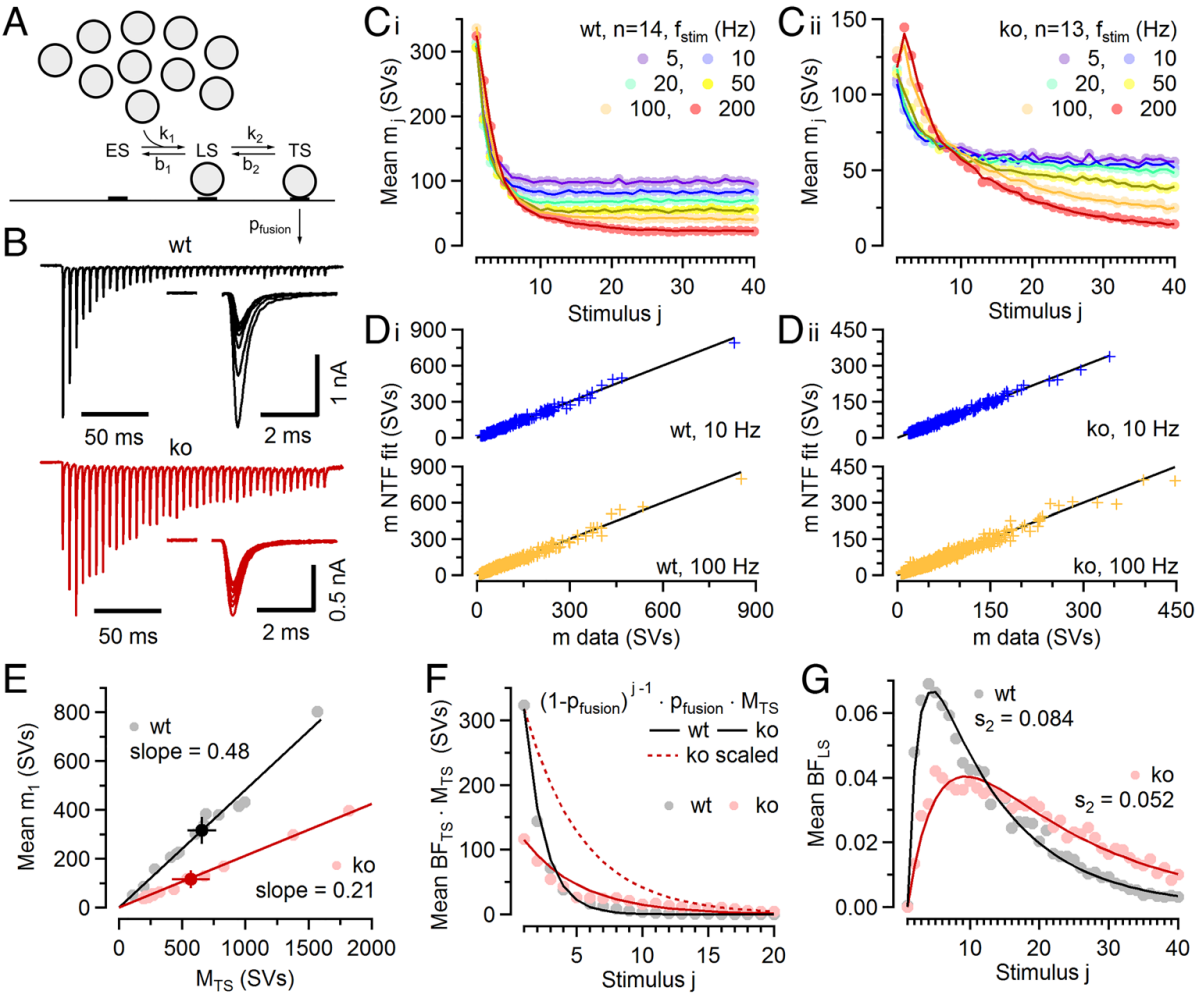
NTF decomposition analysis of EPSC trains recorded in wt and Cplx ko calyx synapses. (*A*) Proposed sequential SV priming and fusion scheme consisting of two reversible priming steps allowing SVs to transition between two distinct priming states referred to as loosely docked (LS) and tightly docked (TS). Only TS SVs are fusion competent. Forward transition rate constants *k*_1_ and *k*_2_ are accelerated by [Ca^2+^]_i_, while backward rate constants are Ca^2+^-independent. Newly recruited SVs which dock to empty sites (ES) are supplied from an infinite replenishment pool. A small fraction of LS SVs transiently converts to TS after each AP and can thereby contribute to subsequent release during high-frequency stimulation (≥50 Hz, compare *SI Appendix*, Fig. S3*A*). (*B*) Sample EPSC trains evoked by 200 Hz and 10 Hz stimulation (40 APs) and recorded in a wt (*black*) and a ko (*red*) synapse. For clarity, only the initial five and the last five responses of the 10 Hz trains are shown superimposed (1 mM of kyn in the bath which reduced quantal size by ~87%). (*C*) EPSC trains obtained from 14 wt synapses (*Ci*) and 13 ko synapses (*Cii*) were subjected to two-component NTF analysis. Timecourses predicted by the NTF-decomposition fits (*lines*) for quantal contents (*m_j_*) during stimulus trains of different frequencies are nearly indistinguishable from the averages of the experimental data (*symbols*). (*D*) Scatter plot of NTF-decomposition fit results vs. experimental data for 10 Hz (*blue*) and 100 Hz (*yellow*) stimulus trains for individual wt (*Di*) and ko (*Dii*) synapses. Each symbol represents a single EPSCs amplitude (average of 3 to 4 repetitions) within the trains recorded in an individual synapse. The data are tightly scattered around the identity line (*black*). (*E*) Scatter plot of initial quantal content (*m*_1_) vs. the size of the preexisting TS pool (*M_TS_*) as obtained by NTF analysis for wt (*gray*) and ko (*red*) synapses. Dark symbols and solid lines represent means and linear regressions, respectively. Regression slopes provide estimates for *p_fusion_*. (*F*) Average timecourses of release contributed by preexisting TS SVs during 5 to 20 Hz EPSC trains were obtained by the product of BF_TS_ · M_TS_ for wt (*gray symbols*) and ko (*red symbols*) synapses. The broken red line represents the ko timecourse scaled to the same initial *m*_1_ as in wt to facilitate comparison. For 5 to 20 Hz EPSC trains, *m_j_* timecourses are well approximated by a geometric series with a constant *p_fusion_* throughout the trains (*solid lines*). (*G*) Average timecourse of BF_LS_ for 5 to 20 Hz EPSC trains in wt (*gray*) and ko (*red*) synapses obtained by three-component NTF decomposition. BF_LS_ represents the normalized timecourse of the contribution of preexisting LS SVs to release. Solid lines represent fits to BF_LS_ with *p_fusion_* constrained to 0.48 and 0.21 for wt and ko synapses, respectively, as previously described (31). These fits yield estimates for the fraction of LS SVs converted to TS SVs per ISI which were 8.4% and 5.2% for wt and ko synapses, respectively.

We first examined Cplx loss-associated changes in AP-induced synchronous release at glutamatergic calyx of Held synapses. To quantify changes in synaptic strength and STP, we employed a recently established kinetic scheme of SV priming and fusion with two distinct SV states, loosely and tightly docked, of which only the latter state is molecularly prepared for fusion (11, 29, 30) (Fig. 1*A*). We recorded excitatory postsynaptic currents (EPSCs) evoked by AP trains of various frequencies (*f_stim_*) in calyx synapses of postnatal day (P) 17 to 21 wild-type (wt) and knock-out (ko) mice (Fig. 1 *B* and *C*). As at this age, Cplx1 is the only paralog detected in calyces and principal MNTB neurons (*SI Appendix*, Fig. S2 *A* and *B*) (6), and Cplx1 ko synapses completely lack Cplx expression. Loss of presynaptic Cplx led to a decrease of initial quantal content (*m*_1_, number of quanta released by an AP) from 317 ± 50 SVs in wt to 117 ± 31 SVs in ko synapses (*P* = 0.001). The decrease in *m*_1_ was accompanied by pronounced changes in STP, where wt synapses depressed strongly during 50 to 200 Hz stimulus trains, while ko synapses often showed prominent initial synaptic facilitation which was followed by depression (Fig. 1 *B* and *C*). For the two-step priming and fusion scheme considered here (Fig. 1*A* and *SI Appendix*, Fig. S3*A*), reduced synaptic strength may result from a lower number of tightly docked SVs (TS SVs) or from lower *p_fusion_* of TS SVs. To differentiate between these two possibilities, which are not mutually exclusive, EPSC trains (5 to 200 Hz) were subjected to two-component nonnegative tensor factorization (NTF) (31) with the aim of deriving estimates for initial *p_fusion_* and the total number of TS SVs in resting synapses (Fig. 1 *C* and *D*). NTF is a “blind source decomposition” technique that decomposes large datasets into linear combinations of a small number of components. As adapted to the analysis of synaptic responses to stimulus trains (31), NTF describes timecourses of quantal release during stimulus trains by the sum of contributions from SV populations, which had been in one of the states of the model at stimulus onset (11, 31). During NTF decomposition, the number of quanta contributed by a given component to the release during trains, e.g., *M_TS_* and *M_LS_* (subscripts representing the vesicle state) is allowed to vary among synapses, while the normalized release timecourses (termed basefunctions [BFs]) are constrained to be identical for all synapses from the same genotype and at the same *f_stim_*. In its simplest form, NTF splits the release timecourse into two components: that of SVs which reside in state TS before stimulation and the rest. TS SVs, represented by basefunction *BF_TS_,* only need to undergo a final fusion step, and are rapidly depleted during repetitive stimulation. The initial value of *BF_TS_* corresponds to the initial *p_fusion_* (*SI Appendix*, Fig. S1*A*) (31). Assuming that stimulus trains are long enough to deplete all preexisting TS SVs, *M_TS_* provides an estimate for the number of TS SVs in individual synapses at rest (31). *M_TS_* varied strongly among synapses (range: 110 to 1,571 SVs [wt], 191 to 1,818 SVs [ko]) but was similar on average, with 656 ± 104 (n = 14) and 566 ± 140 (n = 13) SVs in wt and ko synapses (*P* = 0.62), respectively (Fig. 1*E* and *SI Appendix*, Fig. S1*B*).

Scatter plots of initial quantal content (*m*_1_) vs. *M_TS_* show a strong linear correlation for both wt and ko synapses, however with very different slopes. The slopes of linear regressions to *m*_1_ vs. *M_TS_* plots (i.e. the fractions of TS SVs released by the first AP) reflect the initial *p_fusion_* which was 0.48 and 0.21 for wt and ko synapses, respectively. Because AP-evoked presynaptic Ca^2+^ influx is unchanged following Cplx1 loss (6), this indicates that fusogenicity of TS SVs was robustly reduced by about 56% upon Cplx loss. Thus, the ~63% decreased *m*_1_ in ko synapses is primarily due to a lower fusogenicity of their TS SVs. The marked differences in initial *p_fusion_* (Fig. 1*E* and *SI Appendix*, Fig. S1*A*) are also reflected in the average timecourses of consumption of preexisting TS SVs during 5 to 20 Hz trains (*M_TS_* · *BF_TS_*; Fig. 1*F*). These timecourses are well approximated by assuming a constant fusion probability throughout trains, which yields a simple geometric series $p_{\mathrm{fusion}}\cdot (1-p_{\mathrm{fusion}}{)}^{\;j-1}\cdot M_{\mathrm{TS}}$ , where *j* denotes stimulus number.

Differences between *p_fusion_* of wt and Cplx1 ko synapses, as reported here, were confirmed by the analysis of *PPRs* and steady-state depression described previously (11; their equation 31) and explained in detail in *SI Appendix*.

To establish whether not only *p_fusion_* but also priming kinetics are affected by the loss of Cplx, EPSC train data were subjected to three-component NTF decomposition (11, 31) which yields the normalized timecourse of the contribution of LS SVs to release (*BF_LS_*). As shown before (11, 31), fits to *BF_LS_* yield an estimate for *s*_2_, the fraction of LS SVs undergoing the LS→TS transition during interstimulus intervals (ISIs). Such estimates were 8.4% and 5.2% for 5 to 20 Hz stimulus trains in wt and ko synapses, respectively (Fig. 1*G*), indicating that Cplx loss does not only attenuate *p_fusion_* (Fig. 1 *E* and *F*) but also reduces the stimulus-dependent priming.

**Fig. 2.**
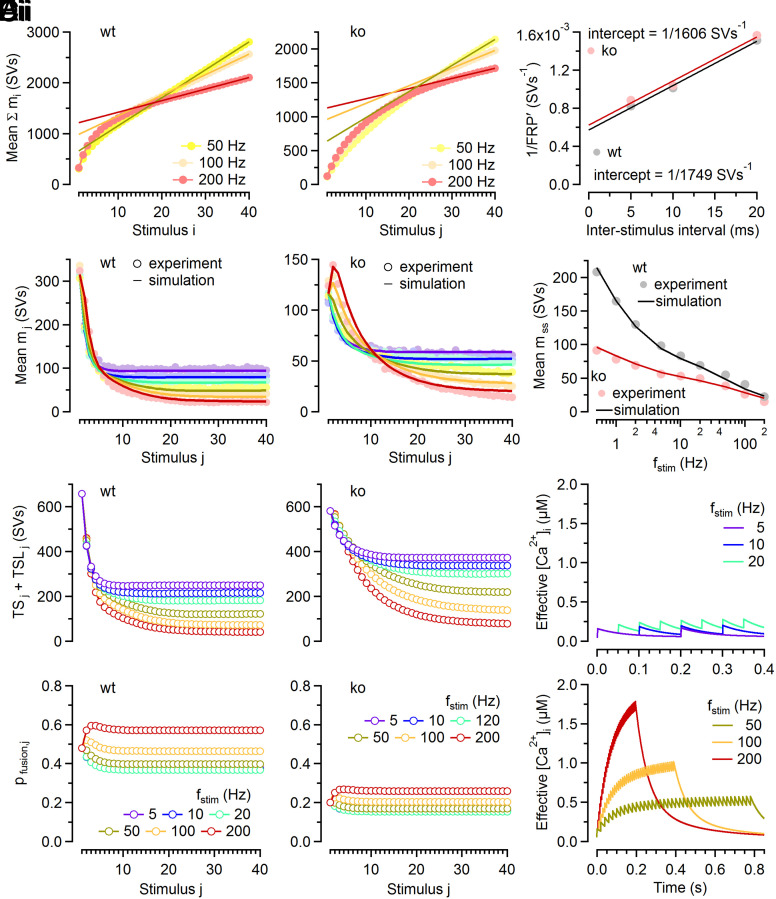
Estimating *FRP* and modeling of STP during AP trains at wt and Cplx ko calyx synapses. (*A*) Mean cumulative quantal contents ( $\sum m_{j}$   ) for *f_stim_* = 50, 100, and 200 Hz in wt (*Ai*) and ko (*Aii*) synapses. Solid lines represent linear regressions fitted to the last five responses and extrapolated to the first stimulus. Intersections of the line fits with the *y*-axis represent apparent pool sizes (*FRP*) for the respective *f_stim_*. (*B*) Scatter plots of 1/*FRP* over ISI for wt (*gray*) and ko (*red*) synapses. Intersections of line fits (*solid lines*) with the *y*-axis represented estimates for 1/*FRP* corrected for incomplete pool depletion. *FRP* estimates (mean ± SEM) were 1,749 ± 308 SVs and 1,606 ± 325 SVs for wt and ko, respectively. SEM estimates were obtained by bootstrap analysis (*SI Appendix*, Fig. S1*C*). (*C*) Comparison of experimental data (*symbols*) and numerical simulations (*solid lines*, using the kinetic scheme in *SI Appendix*, Fig. S3*A*) for the timecourses of *m*_j_ during stimulus trains with *f_stim_* = 5 to 200 Hz in wt (*Ci*) and ko (*Cii*) synapses. (*D*) Comparison of experimental data (*symbols*) and numerical simulations (*solid lines*) for the steady-state release observed for *f_stim_* = 0.5 to 200 Hz in wt (*gray*) and ko (*red*) synapses. (*E*) Model predictions for the changes in the number of fusion-competent SVs (sum of TS and TSL SVs, *Top*) and in *p_fusion_*_,_*_j_* (*Bottom*) during stimulus trains in wt (*Ei*) and ko (*Eii*) synapses. Each symbol represents the value of the respective parameter immediately before stimulus j arrival. (*F*) Model predictions for the timecourse of the “effective” [Ca^2+^]_i_ driving Ca^2+^-dependent acceleration of SV priming.

In order to explore whether loss of Cplx also influences the resting pool size of LS SVs, we determined the sum of LS and TS SVs in resting synapses (Fig. 2 *A* and *B*, and *SI Appendix*, Fig. S1*C*), as previously described (31). Since the resting pool size of TS SVs is known (Fig. 1*E* and *SI Appendix*, Fig. S1*B*), we can readily calculate the resting pool size of LS SVs and found it similar in wt and ko synapses (*SI Appendix*, Fig. S1*D*).

Having established that Cplx loss strongly reduces initial *p_fusion_* and reduces the AP-induced LS→TS transition while leaving the total number of LS and TS SVs at rest largely unaltered, we adjusted the model parameters of the kinetic scheme (*SI Appendix*, Fig. S3*A*) to reproduce experimentally observed average timecourses of quantal release during stimulus trains in wt and ko synapses (Fig. 2*C*). Model predictions for steady-state quantal contents (*m_ss_*) compare well to experimental data (Fig. 2*D*). Predicted timecourse for the number of fusion-competent SVs, *p_fusion_* and the effective [Ca^2+^]_i_ during stimulus trains are shown in Fig. 2 *E* and *F*. Despite constraining differences in model parameters largely to *p_fusion_* and the Ca^2+^-dependence of the second priming step (*SI Appendix*, Table S1), the kinetic scheme reproduced STP and *m_ss_* for a wide range of *f_stim_* (0.5 to 200 Hz) in wt and ko synapses.

Reduced initial synaptic strength in ko synapses could alternatively result from a shift in the balance between LS and TS SVs at rest (11, 32). In addition to contradicting our NTF-based *p_fusion_* estimates, such model variants did not reproduce experimentally observed time course of recovery from synaptic depression (*SI Appendix*, Fig. S4).

In sum, Cplx loss weakens ko synapses to about one third of the strength of wt synapses primarily by decreasing *p_fusion_* of TS SVs, while the sum of LS and TS vesicles in resting synapses remains virtually unaltered. Further, a lower fraction of LS SVs is converted to TS SVs following each AP during low-frequency stimulation in ko as compared to wt synapses.

### Aberrant Delayed Release in Cplx-Deficient Calyx Synapses Is Not Mediated by Syt7.

**Fig. 3.**
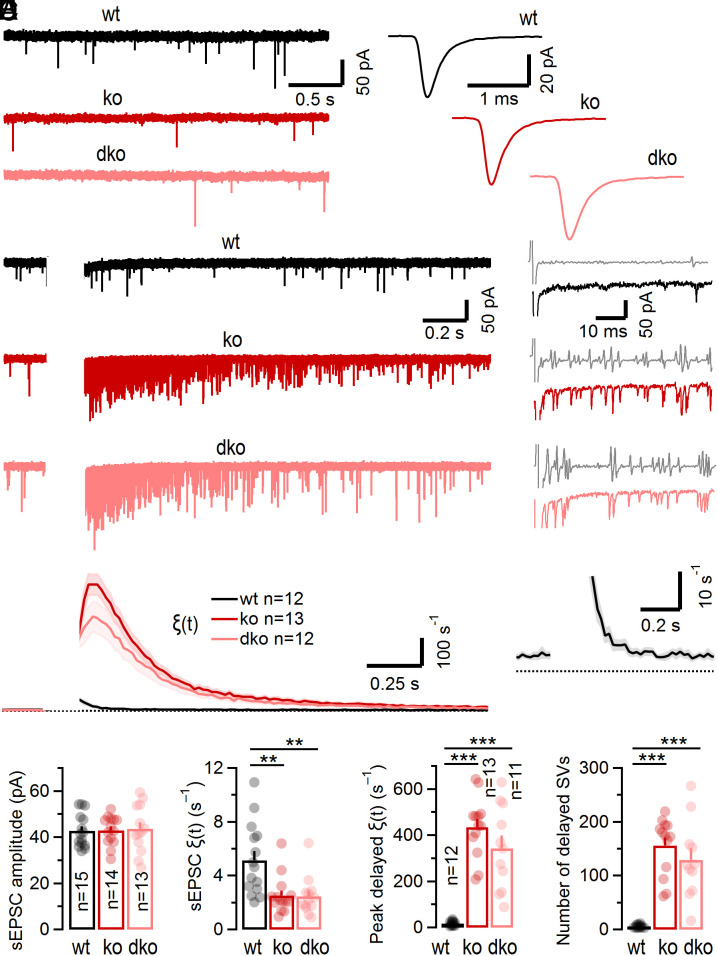
Opposite effects on spontaneous versus delayed quantal release at calyx synapses following genetic ablation of Cplx expression. (*A*) Sample traces (*Left*) and average waveforms (*Right*) of pharmacologically isolated (5 µM strychnine) sEPSCs recorded in a wt (*black*), a Cplx1 ko (*red*), and a Cplx1, Syt7 dko (*light red*) synapse. The recorded sEPSCs likely represent single quanta because afferent axons giving rise to calyces lack spontaneous discharge activity in the slice preparation, and AP-evoked EPSCs are several orders of magnitude larger. (*B*) Sample traces of delayed quantal release following conditioning trains (100 Hz, 15 APs; blanked for clarity) recorded in a wt (*black*), a ko (red), and a dko (*light red*) synapse. Five sweeps are shown superimposed. A single trace and the corresponding low-pass filtered inverted first derivative (*gray*) are shown to the *Right*. The detection of delayed quantal release events was based on a threshold-crossing algorithm. The average timecourse of release *ξ*(*t*) was calculated using a 20 ms bin size. Recording conditions were optimized for resolving individual quanta (absence of kyn from the bath and R_s_ compensation of <50%) resulting in compromised voltage clamp of AP-evoked EPSCs (blanked). (*C*) Averages for *ξ*(*t*) before and after conditioning stimulation in wt (*black*), ko (*red*), and dko (*light red*) synapses. For each synapse, 30 to 100 sweeps were acquired at an intersweep interval of 15 s. Solid lines and shaded areas represent mean and ±SEM, respectively. Delayed release continues to increase after conditioning stimulation in ko synapses. In wt synapses (shown at expanded y-scale in the *Inset*), delayed release was only slightly elevated and decayed monotonically immediately after cessation of stimulation. (*D*) Summary data for sEPSC amplitudes (*Left*) and sEPSC rates (*Right*). Lack of Cplx1 (*red*) or combined genetic ablation of Cplx1 and Syt7 (*light red*) does not affect sEPSC size but strongly attenuates sEPSC frequency. (*E*) Summary data for peak *ξ*(*t*) (*Left*) and total numbers of SVs (*Right*) contributing to delayed quantal release when analyzed over a time period of 1 s starting 10 ms after the last stimulus in the train. Lack of Cplx (*red*) or combined genetic ablation of Cplx1 and Syt7 (*light red*) strongly enhances peak *ξ*(*t*) and total number of asynchronously released quanta. (**P < 0.01; ***P < 0.001).

In addition to reducing AP-evoked synchronous release (Fig. 1), Cplx loss strongly augments delayed release following repetitive stimulation of glutamatergic calyceal brainstem synapses (6, 8). Syt7 binds Ca^2+^ and phospholipids with high affinity (33–35) and has been proposed to mediate asynchronous release (25–27, 36–38). To assess the role of Syt7 in aberrant delayed release seen upon Cplx loss, we compared spontaneous release before and delayed release after stimulus trains in wt, ko, and Cplx1^−/−^/Syt7^−/−^ (dko) synapses (Fig. 3). Syt7 loss in dko mice was confirmed by Western blot analysis (*SI Appendix*, Fig. S5*A*). Immunolabeling of brainstem sections revealed presynaptic anti-Syt7 staining in wt but not in Syt7^−/−^ calyces (*SI Appendix*, Fig. S5*B*).

Consistent with a previous study (6), the rate of spontaneous release events (sEPSCs) measured in ko synapses during a 1 s time period immediately before stimulation was reduced to ~49% of wt levels (from 5.14 s^−1^ to 2.53 s^−1^, *P* = 0.006; Fig. 3 *A* and *D*). The sEPSC frequency in dko synapses (2.45 s^−1^), lacking Syt7 and Cplx, was similar to that seen in only Cplx-deficient synapses and sEPSC amplitudes and kinetics were unaltered in ko and dko synapses (Fig. 3 *A* and *D*). However, analysis of delayed release following 100 Hz conditioning trains revealed a barrage of asynchronous events in ko synapses which was absent in wt but persisted in dko synapses (Fig. 3 *B* and *C*). Given the similarities in spontaneous and delayed release between ko and dko synapses, we conclude that Syt7 does not mediate corresponding changes seen upon Cplx loss.

The timecourse of the quantal release rate (*ξ*(*t*)) before and after train stimulation was quantified at high temporal resolution by counting individual quanta in 20 ms bins using a first derivative–based detection algorithm (Fig. 3*B*). In wt synapses, *ξ*(*t*) increased transiently from ~3 to 6 s^−1^ at rest to a maximum of ~17 s^−1^ immediately following 100 Hz stimulation. This increase in *ξ*(*t*) decayed quickly with a time constant of *τ* ≈ 60 ms (Fig. 3*C*). In ko and dko synapses, the maximum *ξ*(*t*) was >25 times higher (~430 s^−1^) than in wt. Remarkably, *ξ*(*t*) continued to increase even after cessation of stimulation to reach a peak at >60 ms after the end of stimulation and thereafter only slowly decayed back to its basal value (*τ* ≈ 300 ms).

In summary, Cplx loss induces changes in opposite direction with regard to the rates of spontaneous release in resting synapses and delayed release following high-frequency stimulation. In ko synapses, the mean sEPSC frequency was reduced by ~50% (Fig. 3*D*) while peak rates of delayed release and total number of quanta released in a delayed fashion increased by more than an order of magnitude as compared to wt (Fig. 3*E*). We conclude that Syt7 is dispensable for spontaneous release and does not mediate aberrant delayed release upon Cplx loss because dko and ko synapses did not differ in these parameters (Fig. 3 *D* and *E*).

### Aberrant Delayed Release upon Cplx Loss Does Not Represent Fusion of Synchronous Release-mediating TS SVs.

**Fig. 4.**
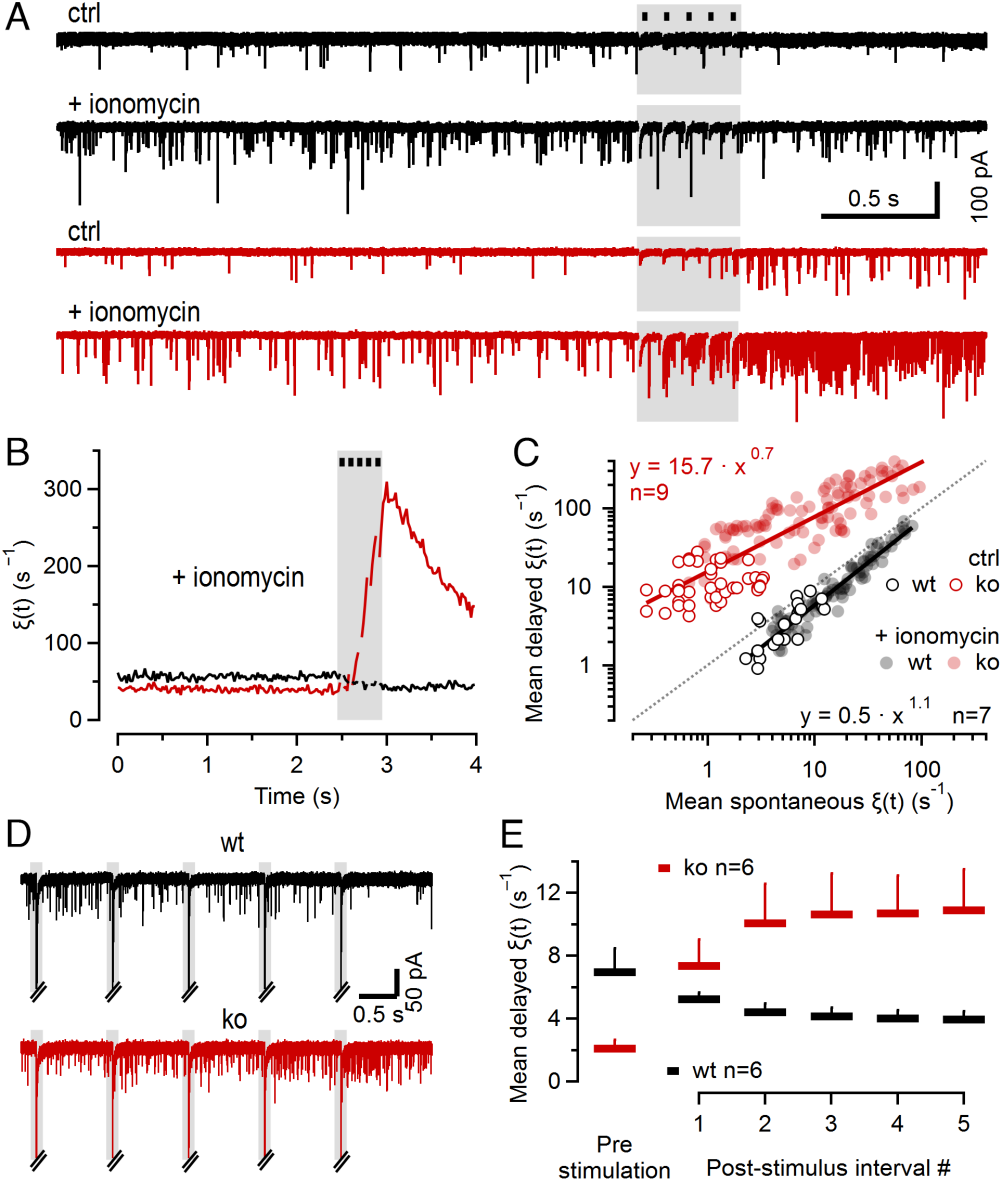
Aberrant delayed quantal release is induced by low-frequency stimulation in Cplx-deficient calyx synapses, in the absence of summation of AP-induced Ca^2+^ transients. (*A*) Sample traces of sEPSCs at rest and following brief conditioning low-frequency stimulation (10 Hz, 5 APs, gray areas) recorded in a wt (*black*) and a Cplx1 ko (*red*) synapse. *Top* and *Bottom* traces illustrate recordings in the absence and presence of 2.5 µM ionomycin, respectively. Synapses were allowed to recover for 15 s after each sweep. Three traces are shown superimposed for the absence of ionomycin (*Top*). (*B*) Averages for *ξ*(*t*) before and after 10 Hz stimulation in wt (*black*, n = 7) and Cplx1 ko (*red*, n = 9) synapses (20 ms binning). In wt synapses, *ξ*(*t*) decreased during stimulation and remained attenuated for ≥1 s. In ko synapses, *ξ*(*t*) transiently increased during and following stimulation before decaying back to resting levels. (*C*) Scatter log–log plot of mean *ξ*(*t*) after 10 Hz stimulation as a function of *ξ*(*t*) before stimulation in wt (*black*) and Cplx1 ko (*red*) synapses. Each symbol represents an average of three consecutive sweeps. Empty symbols and filled symbols represent recordings in the absence and following wash-in of 2.5 µM ionomycin, respectively. Note that wt and Cplx1 ko measurements scatter below and above the identity line (dotted line, indicating same *ξ*(*t*) before and after 10 Hz stimulation), respectively. Solid lines represent linear regressions in log–log space indicating that for *ξ*(*t*) values at rest in the range of 10 to 100 Hz, *ξ*(*t*) following 10 Hz stimulation was reduced to 63 to 79% in wt synapses but enhanced by a factor of 4 to 8 in Cplx1 ko synapses. (*D*) Similar experiment as in (*A*) but using brief 1 Hz stimulus trains (5 APs) to quantify changes in *ξ*(*t*) during ISIs. Sample sweeps recorded in a wt (*black*) and a Cplx1 ko (*red*) synapse. AP-evoked EPSCs are truncated for clarity. While in the wt synapse, *ξ*(*t*) is strongly reduced during the fifth poststimulus interval as compared to the first poststimulus interval, the opposite is observed in the Cplx1 ko synapse. (*E*) Summary data for experiments exemplified in (*D*). A minimum of 20 sweeps was recorded for each synapse. Average values for *ξ*(*t*) before stimulation and during poststimulus intervals are plotted for wt (*black*) and ko (*red*) synapses. The black dots and gray shaded regions in (*A*, *B*, and *D*) indicate the timing of synaptic stimulation. AP-evoked synchronous EPSCs are either blanked (*A* and *B*) or truncated (*D*) for clarity.

The two principal parameters determining delayed release kinetics are i) the availability of fusion-competent SVs and ii) the magnitude and the timecourse of presynaptic global [Ca^2+^]_i_. During ongoing 100 Hz stimulation, these parameters exhibit complex dynamics and the timecourse of delayed release cannot be resolved reliably by counting quanta because individual asynchronous release events can summate or obscured by the prevalence of synchronous AP-triggered release. To examine changes in delayed release with decreasing availability of TS SVs and in the absence of marked changes in presynaptic global [Ca^2+^]_i_, we quantified *ξ*(*t*) during short low-frequency trains (10 or 1 Hz, 5 stimuli, Fig. 4). Summation of AP-induced presynaptic Ca^2+^ transients is expected to be negligible at such low *f_stim_* (39), so that individual Ca^2+^ transients are expected to be largely invariant (Fig. 2*F*) and [Ca^2+^]_i_ following stimulation remains close to resting levels. After collecting 10 4-s sweeps to measure *ξ*(*t*) under control conditions (Fig. 4 *A*, *Top* traces), ionomycin was bath-applied to elevate presynaptic [Ca^2+^]_i_ until *ξ*(*t*) increased to ~100 s^−1^ corresponding to [Ca^2+^]_i_ of ~1 µM (40) (Fig. 4 *A*, *Bottom* traces). Since single APs induce Ca^2+^ transients of <200 nM in mature calyx terminals, 5 stimuli at 10 Hz do not increase [Ca^2+^]_i_ much further. Correspondingly, *ξ*(*t*) in wt synapses did not show a transient increase as observed after 100 Hz stimulation (Fig. 3 *C*, *Inset*), but rather gradually decreased by ~30% after 10 Hz stimulation in the presence of ionomycin, consistent with a reduced availability of TS SVs due to partial pool depletion (Fig. 4*B*). In contrast, *ξ*(*t*) increased >sevenfold following 10 Hz stimulation of ko synapses despite a reduced availability of TS SVs and despite a nearly constant [Ca^2+^]_i_ (Fig. 4*B*). When plotting mean *ξ*(*t*) values obtained after stimulation (mean over 1 s after 10 Hz train) as a function of those measured before stimulation (mean over 2.5 s before 10 Hz train) at various time points following ionomycin wash-in, wt, and ko data were clearly separated and scattered below and above the identity line, respectively (Fig. 4*C*).

To measure how *ξ*(*t*) changes during ongoing stimulation we further reduced *f_stim_* to 1 Hz (Fig. 4*D*). In wt synapses, the mean *ξ*(*t*) gradually decreased from ~7 s^−1^ at rest to ~4 s^−1^ after the fifth stimulus (Fig. 4*E*). This ~40% decrease in delayed release likely reflects partial depletion of fusion competent TS SVs and is comparable to the average decrease in synchronous release during 1 Hz stimulation (1 − *m_ss_*/*m_0_* = 1 − 165 SVs/310 SVs ≈ 47%; Fig. 2*D*). In contrast, *ξ*(*t*) increased substantially in ko synapses, from ~2 s^−1^ at rest to ~11 s^−1^ after the fifth stimulus (Fig. 4*E*), while synchronous release decreases (1 − *m_ss_*/*m_0_* = 1 − 80 SVs/120 SVs ≈ 33%) as seen in wt synapses. Thus, the timecourse of *ξ*(*t*) during 1 Hz stimulation showed opposite trends in wt and ko synapses while the relative decrease in TS SV-mediated synchronous AP-evoked release was comparable.

Taken together, we conclude that the fivefold-to-sevenfold increase in delayed release following brief 1 or 10 Hz stimulation of Cplx-deficient synapses is incompatible with the assumption that aberrant delayed release represents fusion of TS SVs with a possibly altered Ca^2+^ sensitivity because of the following considerations: i) Presynaptic [Ca^2+^]_i_ transients do not summate during low-frequency stimulation but remain invariant throughout the trains, ii) consequently, presynaptic [Ca^2+^]_i_ after stimulus trains is close to resting levels, iii) *p_fusion_* is expected to remain nearly constant during such low stimulation frequencies, and iv) the number of TS SVs is expected to decrease as indicated by the measured *m_ss_* during 1 or 10 Hz stimulation (Fig. 2*D*). These arguments together with the observation that delayed release in ko synapses continued to increase even after cessation of 100 Hz stimulation while residual [Ca^2+^]_i_ already decays back to resting levels (Figs. 2*F* and 3*C*) prompted us to explore alternative mechanisms that may account for aberrant delayed release in Cplx-deficient synapses.

### A Kinetic Priming Scheme with a Faulty Vesicle State Reproduces the Delayed Release Phenotype Caused by Cplx Loss.

**Fig. 5.**
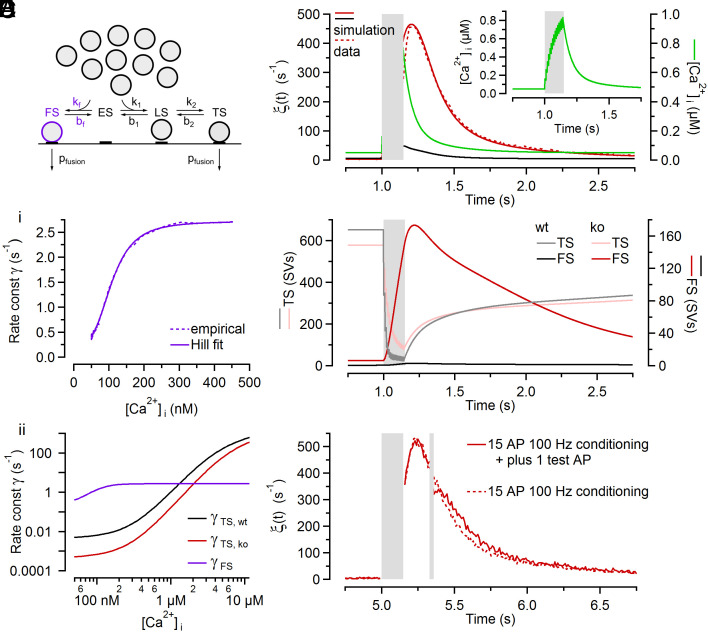
A kinetic scheme with a faulty SV state reproduces the timecourse of aberrant delayed release observed in Cplx-deficient calyx synapses. (*A*) Proposed extension for the kinetic scheme shown in Fig. 1*A* by adding a branch to a FS. (*B*) Comparison of numerical simulations and experimental data for spontaneous and delayed *ξ*(*t*) following conditioning stimulation (100 Hz, 15 APs) for wt (*black*) and ko (*red*) synapses. The timecourse of the effective global [Ca^2+^]_i_ is shown for comparison (*green*) on the right axis and in the *Inset*. (*C*) Ca^2+^ dependence of fusion for FS SVs (*Ci*, *purple*) in comparison to TS SVs in wt (*Cii*, *black*) and ko (*Cii*, *red*) synapses. The dynamic range of the Ca^2+^ dependence of fusion is greatly reduced for FS SVs. (*D*) Timecourses of occupancy of FS (*dark lines*, *right axis*) and TS (*pale lines*, *left axis*) in wt (*black*) and ko (*red*) synapses. The period of 100 Hz conditioning stimulations is indicated by the gray box. (*E*) The timecourse of decay of the delayed quantal release following conditioning stimulation (100 Hz, 15 APs; *left gray box*) in ko synapses is largely unchanged when delivering an additional test AP (*right gray box*).

We hypothesized that Cplx loss increases the propensity of SVs to associate with a release apparatus in a faulty molecular configuration and explored whether a small number of docked and primed SVs in a faulty state (FS) may be sufficient to account for aberrant delayed release in Cplx-deficient synapses. Although likely virtually absent in resting wt and ko synapses, the number of faulty SVs (FS SVs) may transiently increase during AP activity in Cplx-deficient synapses because elevated [Ca^2+^]_i_ stimulates vesicle priming (41). To explore such a mechanism by mathematical modeling, we extended the sequential two-step priming scheme (Fig. 1*A* and *SI Appendix*, Fig. S3*A*) by a faulty vesicle state (Fig. 5*A* and *SI Appendix*, Fig. S3*B*). In analogy to the ES↔LS and LS↔TS transitions, we assumed that only the forward rate constant of the ES↔FS transition (*k_f_*) is Ca^2+^ dependent but not its backward rate constant (*b_f_*). The parameters *k_f_* and *b_f_*, and the Ca^2+^ sensitivity of FS SV fusion were adjusted to reproduce experimental observations while all other model parameters were identical to those of the initial model without the FS (Fig. 2 *C* and *D*). Numerical simulations based on the extended kinetic scheme (*SI Appendix*, Fig. S3*B*) reproduced timecourse and magnitude of *ξ*(*t*) for wt and ko synapses (Fig. 5*B*). Presynaptic global [Ca^2+^]_i_, which was assumed to increase instantaneously after AP arrival and to decay with a double-exponential timecourse (22, 24, 39, 42), is shown for comparison (Fig. 5*B*, *Inset*). The model prediction for the relationship between [Ca^2+^]_i_ and fusion rate constant of FS SVs is well fit by a Hill function with a maximum fusion rate constant (*γ*) of ~2.72 s^−1^, a Hill coefficient n of ~3.4 and an apparent K_D_ of ~107 nM (Fig. 5*Ci*). At resting [Ca^2+^]_i_ (50 nM), the postulated fusogenicity of FS SVs (*γ* ≈ 0.40 s^−1^) is over two orders of magnitude higher than that of TS SVs in ko synapses, but increases only about sevenfold when raising [Ca^2+^]_i_ levels from 50 nM to ≥0.5 µM (Fig. 5*Cii*). As expected, the simulation predicts strong depletion of TS SVs during a 100 Hz train for wt and also for ko synapses, albeit with a slower timecourse for the latter because of their lower *p_fusion_* (Fig. 5*D*). While the total number of FS SVs remains small (≤12 SVs) during and after 100 Hz stimulation in wt synapses, their number increases strongly and transiently in ko synapses (Fig. 5*D*) competing with LS and TS SVs for a fixed number of vesicle docking sites (*N_tot_* = 1,900; *SI Appendix*, Table S1). At the time of maximum FS occupancy (~170 SVs), presynaptic global [Ca^2+^]_i_ has decayed to ~340 nM (Fig. 5*B*). This [Ca^2+^]_i_ level corresponds to a FS SV fusion rate constant of ~2.7 s^−1^ (Fig. 5*Ci*). Accordingly, a maximum *ξ*(*t*) of approximately 170 · 2.7 s^−1^ = 450 s^−1^ is generated in ko synapses.

To experimentally validate our assumption that the [Ca^2+^]_i_-fusion relationship of FS SVs has a small dynamic range (Fig. 5*C*) and, specifically, that their fusion rate constant does not increase substantially for [Ca^2+^]_i_ levels above 0.5 µM, we employed a stimulus protocol consisting of a conditioning 100 Hz train (15 stimuli) followed by a single test stimulus 200 ms later when delayed release is elevated in ko synapses (Fig. 5*E*). Since the timecourse of delayed release was similar in ko recordings with and without the test stimulus, the fraction of FS SVs consumed in response to the test stimulus must be negligible, indicating that *p_fusion_* of FS SV is low. This corroborates our assumption that the fusogenicity of FS SVs at high [Ca^2+^]_i_, as predicted for local [Ca^2+^]_i_ domains triggering vesicle fusion during AP firing ([Ca^2+^]_i_ ≫ 10 µM) (43–46), is much lower than that of TS SVs.

In sum, a kinetic SV priming and fusion scheme including a faulty SV state reproduces AP-evoked synchronous, and spontaneous and delayed release before and after synaptic stimulation, respectively, in wt and ko synapses. The FS occupancy is assumed to be low in resting wt and ko calyces, but increases transiently during and immediately after high-frequency stimulation in the absence of Cplx. At any time during and after stimulation, the maximum number of SVs residing in the FS did not exceed 170 which is less than a third of the number of TS SVs in resting wt and ko synapses. At resting [Ca^2+^]_i_, the probability of an FS SV to fuse is more than two orders of magnitude higher than that of a TS SV. However, the fusion rate constant of faulty SVs increases by only one order of magnitude at high [Ca^2+^]_i_ while the fusogenicity of TS SVs increases by more than six orders of magnitude when increasing [Ca^2+^]_i_ from 50 nM to 50 µM.

### Model Predictions for Changes in Spontaneous Release upon Cplx Loss Depend on the ES→FS Transition Kinetics at Resting [Ca^2+^]_i_.

**Fig. 6.**
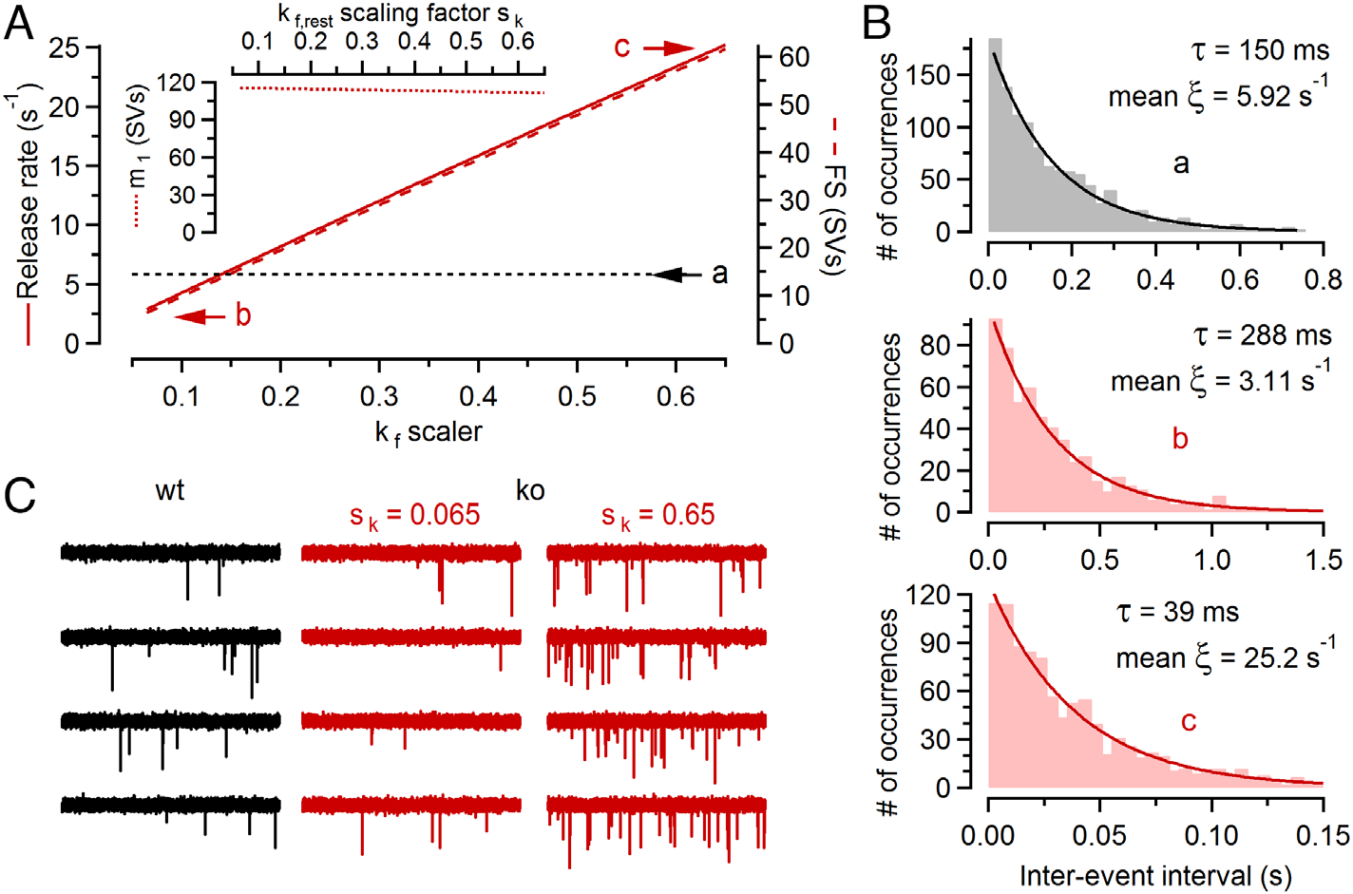
The predicted spontaneous release for Cplx-deficient synapses strongly depends on the ES→FS transition kinetics at resting [Ca^2+^]_i_. (*A*) Spontaneous release rate (*solid line, left axis*) and occupancy of FS (*broken line, right axis*) in resting ko synapses and *m*_1_ (*Inset*) plotted as a function of the *k_f,rest_* scaling factor *s_k_* ( $k_{f,rest}=s_{k}\mathrm{Ã‚}\mathrm{Â\cdot}k_{1,rest}$   ; *SI Appendix*, Table S1). The dotted line labeled with a indicates the spontaneous release rate in wt synapses. The letters b, c designate results obtained with different *k_f,rest_* scaling factors (*s_k_* = 0.065 and *s_k_* = 0.65). (*B*) MC simulations of spontaneously occurring quantal release using the same model parameters as in Fig. 5*B*, except for the *k_f,rest_* scaling factor for the conditions designated with c which was increased 10 times (*s_k_* = 0.65). 1,185, 623 and 1,008 release events were simulated for condition a, b, c, respectively. The distributions of interevent intervals decayed exponentially. Solid lines represent exponential fits which yielded decay time constants that were similar to the reciprocal of the corresponding mean *ξ*(*t*). (*C*) Individual sample traces for the MC simulations shown in (*B*). The spontaneous release rate *ξ*(*t*) in resting ko synapses is lower (*Middle*) or higher (*Right*) than the corresponding value in wt (*Left*) synapses depending on the value of *k_f_* at resting [Ca^2+^]_i_. For display purposes, event amplitude distributions were modeled with a beta distribution to mimic experimentally observed mean amplitudes and skewed distributions.

At many vertebrate synapses, loss of Cplx leads to reduced spontaneous release rates (3, 4, 6, 8, 19), consistent with the notion that Cplx acts as a fusion promoter. However, increased rates of spontaneous release were observed at Cplx-deficient invertebrate synapses (47–49), lending support to the notion that Cplx “clamps” vesicle fusion (50, 51). The seemingly contradictory phenotypes observed upon Cplx loss in different species prompted us to study whether our model provides a mechanism to reconcile the conflicting experimental results. We explored model predictions for spontaneous release depending on the choice of the ES→FS transition rate constant (*k_f,rest_*) in resting ko synapses (Fig. 6*A*) which may, in principle, differ between different model species and synapse types. Varying *k_f_* over a 10-fold range hardly affected the number of TS SVs in resting ko synapses and therefore left their initial synaptic strength (*m*_1_) virtually unaltered (Fig. 6 *A*, *Inset*). However, the number of FS SVs in resting ko synapses increased from <10 SVs (*k_f_* = 0.065 · *k_1,rest_*) to >60 SVs (*k_f_* = 0.65 · *k_1,rest_*) which resulted in simulated rates of spontaneous release either slightly below or substantially above those measured in wt synapses (Fig. 6*A*). To corroborate these deterministic model predictions, we performed Monte-Carlo (MC) simulations of spontaneous release and analyzed interevent intervals for ~600 to ~1,100 simulated fusion events (Fig. 6 *B* and *C*). As expected for a Poisson process, histograms of the intervals showed exponential distributions with time constants very close to the predicted mean spontaneous release rates (Fig. 6*B*). As with the deterministic model approach (Fig. 6*A*), MC simulations yielded mean spontaneous release rates that were either lower or substantially higher than that in wt synapses depending on the choice for *k_f,rest_* (Fig. 6 *B* and *C*).

In conclusion, depending on the rate constant of the ES→FS transition in resting synapses, attenuation or augmentation of spontaneous release is predicted for Cplx-deficient synapses. Thus, our kinetic model reproduces the inhibitory effect of Cplx loss on synchronous AP-triggered release, and has the intriguing capability to predict either decreased or increased spontaneous release, both of which were observed following Cplx loss in different model species and synapse types.

### Aberrant Delayed Release Is a Widespread Feature of Cplx-Deficient CNS Synapses.

So far, our data describe the magnitude and timecourse of *ξ*(*t*) following conditioning stimulation at ko and dko in comparison to wt calyx synapses (Fig. 3 *B* and *C*). Together with endbulb of Held synapses and the neuro-muscular junction, calyx of Held synapses constitute a group of exceptionally large excitatory synapses with conspicuously augmented delayed release upon Cplx loss (6, 8, 19). To assess whether other synapse types exhibit similar functional deficits upon Cplx deficiency, we next examined two types of small inhibitory CNS synapses of the brainstem and the cerebellum which - like calyx synapses - were found to solely express the Cplx1 paralog (*SI Appendix*, Figs. S6 and S7). Glycinergic IPSCs were elicited by afferent fiber stimulation of MNTB→LSO synapses and delayed glycine release was quantified in LSO PNs of wt, ko, and dko mice (*SI Appendix*, Fig. S6 *A*–*F*), much like described above for calyx synapses. In sections prepared from Cplx1^−/−^ mice, MNTB PN cell bodies (*SI Appendix*, Fig. S2 *A* and *B*) and VIAAT-positive boutons surrounding LSO PNs, likely representing glycinergic MNTB inputs, lacked Cplx1,2 immunostaining (*SI Appendix*, Fig. S2 *C* and *D*). Syt7 expression in glycinergic terminals contacting LSO PNs of wt mice was confirmed by anti-Syt7 immunolabeling using sections from Syt7^−/−^ mice as negative controls (*SI Appendix*, Fig. S7 *A* and *B*). Delayed release was enhanced in the absence of Cplx in ko and dko MNTB→LSO synapses (*SI Appendix*, Fig. S6 *C* and *D*), confirming our earlier conclusion that aberrant delayed release in Cplx-deficient synapses does not require Syt7. On average, the peak of *ξ*(*t*) and the total number of delayed quanta released after conditioning 100 Hz stimulation were more than fourfold larger in ko and dko as compared to wt synapses (*SI Appendix*, Fig. S6*F*). By contrast, the frequency of spontaneous IPSCs (sIPSCs) was strongly reduced in ko and dko MNTB→LSO synapses. sIPSC amplitudes were unaltered (*SI Appendix*, Figs. S6*E* and S7*C*). GABAergic molecular layer interneuron to Purkinje cell (MLI →PC) synapses, which also solely express the Cplx1 paralog (*SI Appendix*, Fig. S2 *E* and *F*) and coexpress Syt7 (*SI Appendix*, Fig. S7*D*) presynaptically (26), showed similar aberrations of transmitter release modes as seen earlier in glutamatergic calyx synapses and glycinergic MNTB→LSO synapses (*SI Appendix*, Figs. S6 *G–L* and S7*E*).

In conclusion, aberrant delayed release and attenuation of spontaneous release upon Cplx loss is not specific to large glutamatergic synapses of the mouse CNS, but also observed at small inhibitory glycinergic and GABAergic synapses.

## Discussion

We combined electrophysiological and mathematical modeling approaches to mechanistically dissect the effects of Cplx loss on spontaneous, AP-evoked synchronous, and delayed asynchronous transmitter release at three synapse types in the mouse brain. We consistently observed reduced synaptic strength upon Cplx-deletion, but noticed contrasting effects on spontaneous release in resting synapses and on delayed release following conditioning stimulation. Specifically, the rates of spontaneous release were reduced by >50% in the three Cplx-deficient synapses whereas delayed release was conspicuously augmented. To determine the mechanisms underlying these seemingly contradictory results, we employed the recently proposed (29, 30) and validated (11, 32) sequential two-step vesicle priming scheme, extended by a faulty vesicle state. Model parameters were adjusted based on the NTF decomposition analysis of EPSC trains in wt and ko calyx of Held synapses (Fig. 1). Numerical simulations reproducing spontaneous, evoked, and delayed release led to the following four main conclusions:

(1) A sequential two-step priming scheme reproduces timecourses of AP-evoked synchronous release during stimulus trains over many stimulation frequencies in wt and Cplx-deficient synapses, the latter exhibiting profoundly reduced initial synaptic strength and altered STP.

(2) Cplx-loss induces a ~56% reduction in *p_fusion_* and slows the speed of the transition from the loosely to the tightly docked SV state while the total number of primed SVs remains unchanged.

(3) Augmented delayed release in Cplx-deficient synapses can be accounted for by fusion events originating from a faulty vesicle state which is sparsely populated at rest. During and after presynaptic activity, the number of faulty SVs increases transiently in Cplx-deficient synapses competing with LS and TS SVs for a fixed total number of release sites.

(4) Depending on the parameter values defining the transition kinetics from the undocked (ES) to the faulty (FS) SV state at rest, either attenuated or enhanced spontaneous quantal release can result in Cplx-deficient synapses.

In conclusion, we present a universal and comprehensive model of Cplx’s function in SV priming and fusion that reproduces spontaneous, AP-induced, and delayed release phenotypes in wt and Cplx-deficient synapses.

### Reduction of *p_fusion_* upon Cplx Loss Attenuates Synaptic Depression and Promotes Net Facilitation.

Cplx deletion reduced synaptic strength at calyx synapses by ~63%, consistent with the notion that Cplx acts primarily as a release-promoting factor at conventional mammalian synapses (3, 4, 6, 8, 9). In the sequential two-step priming scheme, the amount of AP-evoked transmitter release is defined by two principal parameters, i) the probability of TS SVs to fuse in response to an AP (*p_fusion_*), and ii) the probability of a given release site being occupied by a fusion-competent TS SV. Because conventional methods for measuring the size of the readily releasable SV pool report a quantity that is close to the sum of LS and TS SVs at calyx synapses (11), we subjected ensembles of EPSC trains to NTF decomposition analysis to obtain estimates for *p_fusion_* and the number of TS SVs (Fig. 1). NTF analysis indicated that Cplx loss primarily decreases *p_fusion_* while leaving the number of primed SVs unaltered (3, 4, 6, 9). Reduced *p_fusion_* in Cplx-deficient synapses slows TS SV consumption during high-frequency stimulation thereby converting paired-pulse depression to paired-pulse facilitation at *f_stim_* ≥ 50 Hz. Numerical simulations using NTF-derived model parameters accurately reproduced changes in initial quantal contents (*m*_1_) and STP during trains upon Cplx loss, and closely predicted steady-state quantal contents (*m_ss_*) for *f_stim_* ranging from 0.5 to 200 Hz (Fig. 2) in wt and ko synapses.

### Enhanced Delayed Release in Cplx-Deficient Synapses Does Not Require Syt7 and Is Unlikely to Originate from Tightly Docked SVs.

We observed strongly enhanced delayed release upon Cplx deletion at calyx of Held synapses (Figs. 3 and 4), glycinergic MNTB→LSO synapses and GABAergic MLI→PC synapses alike (*SI Appendix*, Fig. S6). As these synapses only contain the Cplx1 paralog (*SI Appendix*, Fig. S2), they are ideally suited to assess effects of complete Cplx elimination in situ by using viable Cplx1^−/−^ mice which express other synaptic proteins at normal levels (9). Further, all aforementioned synapse types also express Syt7 (*SI Appendix*, Figs. S5 and S7), whose high apparent Ca^2+^ affinity and slow phospholipid dissociation kinetics make it a prime candidate Ca^2+^ sensor in asynchronous release (33, 52). Syt7 deletion attenuates asynchronous transmitter released at some synapses (25–27, 36–38), whereas other synapses show persistent delayed release, albeit with altered kinetics (53). We therefore tested in all three of our model synapses whether the aberrant delayed release upon Cplx loss requires Syt7 by genetic ablation of Syt7 in Cplx1^−/−^ mice (dko), but did not obtain any evidence for an involvement of Syt7.

Interestingly, deletion of Syt1 and/or Syt2 potentiates asynchronous release (53–58) possibly due to a lack of a suppression of premature SV fusion, indicating that Syt1/Syt2 can “clamp” SV fusion at low [Ca^2+^]_i_ (59). Alternatively, asynchronous release might increase in Syt1/Syt2-deficient synapses because the core SV release machinery incorporates alternative Ca^2+^ sensing proteins that are excluded from triggering SV fusion in the presence of Syt1/Syt2. While we can reject reduced Syt1 and/or Syt2 protein levels as the principal cause of aberrant delayed release upon Cplx loss because Syt1- and Syt2-expression is not reduced (*SI Appendix*, Fig. S5), it is possible that Cplx binding stabilizes Syt1/Syt2-SNARE interactions which are therefore weakened in Cplx-deficient synapses to allow other Ca^2+^ sensors to replace Syt1/Syt2.

### Numerical Simulations Assigning Cplx the Role of a Checkpoint Protein in Fusion-Machinery Assembly Reproduce Delayed Release Phenotypes in Cplx-Deficient Synapses.

Our data so far rule out an involvement of Syt1, Syt2, or Syt7 in the enhancement of delayed release upon Cplx deletion. Further, it is unlikely that Cplx loss alters presynaptic Ca^2+^ dynamics resulting in higher or longer lasting residual [Ca^2+^]_i_ transients that cause aberrant delayed release, because Cplx-deficient calyces do not show altered Ca^2+^ influx (6). Finally, it is implausible that aberrant delayed release in Cplx-deficient synapses is caused by a profound change in the [Ca^2+^]_i_-transmitter release relationship of the SV fusion machinery. This relationship is altered by some presynaptic perturbations (56, 60), and Cplx preferentially binds with high affinity to assembled SNARE complexes and is an essential constituent of the release apparatus at conventional synapses (2, 59, 61, 62). However, to account for all experimental findings related to Cplx loss, it would have to alter the [Ca^2+^]_i_-transmitter release relationship in a very complex fashion, leading to decreased spontaneous SV fusion at low, augmented asynchronous release at intermediate, and lower AP-evoked synchronous release at high [Ca^2+^]_i_ levels.

In view of the arguments above, we considered the alternative scenario that Cplx might act as a checkpoint protein that ensures the correct assembly of the fusion machinery, so that in its absence the checkpoint is leaky and docked SVs increasingly establish a release apparatus in a faulty molecular configuration (63) that exhibit an aberrant [Ca^2+^]_i_-transmitter release relationship. To this end, we simulated delayed release with the two-step sequential priming scheme, extended by a faulty vesicle state, and postulated the ES↔FS transition to be Cplx-dependent. For resting and residual [Ca^2+^]_i_ levels, the fusogenicity of SVs residing in the FS was assumed to be elevated in comparison to SVs associated with a properly assembled release apparatus. Because spontaneous fusion rates are reduced after Cplx loss in the synapses studied here and at most conventional mammalian CNS synapses tested, SVs with a faulty release apparatus are expected to be scarce at rest even in Cplx-deficient synapses. The transiently increased abundance of faulty SVs during and after presynaptic discharge activity leads to aberrantly increased delayed release with a timecourse that closely matches experimental observations.

Importantly, our simulations require only a small minority of SVs to reside in the FS in resting synapses (<0.4% of all docked SVs). When priming activity is augmented during presynaptic activity, more SVs are diverted from the LS→TS priming pathway and transiently accumulate in the FS in ko synapses because of the Ca^2+^-dependence of the ES→FS transition which was assumed to be identical to that of the ES→LS transition (*SI Appendix*, Table S1). The total number of SVs undergoing delayed release in ko synapses is only ~150, which is comparable to the mean quantal content of their initial EPSC. Thus, the number of delayed fusion events is too small to effectively compete with synchronous release and substantially affect STP during short stimulus trains in ko synapses. However, this may not be the case for longer high-frequency trains. Interestingly, while initial synaptic strength can be rescued in ko synapses with elevated external [Ca^2+^], their steady-state release during relatively long 100 Hz trains gradually declines and nearly vanishes (6; their figure 6D2). This can be explained by a high occupancy of the FS under such conditions which diverts a majority SVs from the ES→LS→TS priming pathway but contributes little to synchronous AP-induced due to the small dynamic range of the relationship between [Ca^2+^]_i_ and fusion rate constant for FS SVs.

### The Nature of the Faulty Fusion Machinery.

The notion of a faulty assembly of the release apparatus is supported by the following observations: i) When soluble SNARE protein fragments interact in solution, improperly assembled SNARE-complex configurations occur (64), demonstrating that the assembly of the SV fusion apparatus is not fault-free; ii) the SNARE interactor Munc18 catalyzes SNARE-complex assembly by forming an intermediate template complex, so that Munc18 loss increases the probability of misassembled SNARE complexes (65); iii) Cplx inhibits SNARE-complex formation (66), promotes proper molecular configurations (67), and stabilizes assembled SNARE complexes in vitro (66), indicating that it steers the underlying molecular assembly process.

Because of their elevated fusogenicity at resting and residual [Ca^2+^]_i_ levels, FS SVs may, phenomenologically, be regarded “unclamped” (50, 51, 68–70). An intriguing feature of our model is its ability to generate both, enhanced (apparently unclamped) and attenuated (apparently “superclamped”) spontaneous release in Cplx-deficient synapses depending on a single model parameter (*k_f,rest_*). Mechanistically, Cplx does not act as a fusion clamp in this model but rather as a checkpoint protein that controls the assembly of the release machinery and thus prevents SVs from entering a faulty primed state with aberrant molecular configuration—by precluding the assembly of a faulty release apparatus (i.e., reducing *k_f_*) or by promoting its disassembly (i.e., increasing *b_f_*). Cplx1 prevents disassembly of “properly assembled” trans-SNARE complexes by NSF and αSNAP as monitored in vitro using a FRET assay (71), consistent with a role for Cplx in steering assembly/disassembly of the release machinery in addition to that in controlling SV fusogenicity.

In PC12 cells expressing Cplx and truncated versions of SNARE proteins, Cplx can bind to mutant SNARE complex versions that mimic partially assembled stages, and the SNARE complex affinity of Cplx correlates positively with the extent of SNARE complex preassembly. These results indicate that Cplx can bind to SNARE complexes before their complete formation (72) and may, therefore, act as an adaptor or checkpoint protein that controls the association of the release apparatus (63).

Cplx and Syt1 were proposed to bind to SNARE complexes in a coordinated fashion and to thereby clamp fusion (73). Recent models of the Cplx-Syt-SNARE complex, combining crystal-derived (74, 75) and NMR-based protein complex structures (73, 76) with molecular dynamics simulations (77), envision a scenario where Cplx and Syt1 bind to distinct sites on the SNARE complex. Cplx-Syt-SNARE interactions would be sterically confined to the limited space between a docked vesicle and the plasma membrane, and it is possible that in the absence of Cplx, Syt1 or Syt2 interact differently with the SNARE complex (78) or that Ca^2+^ sensors other than Syt1 or Syt2 engage, thereby changing the Ca^2+^ sensitivity of the fusion process. This possibility would also apply if Cplx and Syt1 bound to the SNARE complex at a shared interface in a tripartite complex (79).

Sixteen Syts are expressed in the brain, eight of which bind Ca^2+^ (2, 80, 81). Besides Syt2, which mediates AP-evoked synchronous release (56, 60), mature calyx terminals contain Syt3 and Syt7 (82, 83). We considered the possibility that SVs may preferentially associate with Syt7 when Cplx is deleted but found delayed release to be similar in Cplx-deficient synapses and synapses lacking both Syt7 and Cplx.

Even though the experimental data and numerical simulations presented here do not allow us yet to identify the specific molecular configuration of the faulty vesicle state, we propose, they demonstrate that all presynaptic phenotypes associated with Cplx loss—augmented or reduced spontaneous release, reduced AP-evoked release, and aberrant delayed release—can be reproduced by our faulty vesicle state model without assigning Cplx the mechanistic role of a “fusion clamp.” In our view, therefore, the mechanism proposed here reconciles the existing conflicting interpretations of the role of Cplx in vesicle fusion which derive from seemingly incompatible observations in different model organisms and synapse types.

## Materials and Methods

### Mouse Lines and Slice Preparation.

Juvenile, posthearing onset (P15–21) mice of either sex derived from the lines C57/Bl6-Cplx1 (9) and C57/Bl6-Syt7 (84) were used. All experiments complied with the German Protection of Animals Act and with the guidelines for the welfare of experimental animals issued by the European Communities Council Directive. Acute brainstem slices were prepared similarly as previously described (39). See *SI Appendix* for details.

### Electrophysiology.

Whole-cell patch-clamp recordings were made from MNTB PNs, LSO PNs or cerebellar MLIs and PCs. EPSCs and IPSCs were pharmacologically isolated. See *SI Appendix* for details.

### Decomposition of Quantal Release into Distinct Components Using NTF.

NTF of EPSC trains was performed similarly as previously described (11, 31). See *SI Appendix* for details.

### Kinetic Scheme for SV Priming and Fusion.

Timecourses of AP-evoked synchronous release during stimulus trains were simulated using a sequential two-step priming scheme as previously described (11). The kinetic model was solved for steady-state at the beginning of the simulation and then allowed to evolve by numerical integration. To simulate the time-resolved rate of spontaneous and delayed release [*ξ*(*t*)] simultaneously with discrete AP-evoked synchronous release events, *ξ*(*t*) was calculated before stimulation, during interstimulus intervals and after stimulation. See *SI Appendix* for details.

### Immunostaining, Confocal Microscopy, and Image Analysis.

The following primary Abs were used: rabbit anti-Cplx1/2 (1:500; #122 002, Synaptic System), guinea pig anti-vesicular glutamate transporter 1 (VGLUT1, 1:500; #135 304, Synaptic System), guinea pig anti-vesicular GABA/Gly transporter (VGAT, 1:500; #131 004, Synaptic System), and rabbit anti-Syt7 (1:500; #105 172, Synaptic System). Images were acquired on a Leica SP2 confocal microscope (Leica Microsystems). Image analysis was carried out using ImageJ (NIH). See *SI Appendix* for details.

## Supplementary Material

Appendix 01 (PDF)

## Data Availability

Igor program code data have been deposited in zenodo (https://doi.org/10.5281/zenodo.6818173) (85).
