# Supplementary material for: Complexin has a dual synaptic function as checkpoint protein in vesicle priming and as a promoter of vesicle fusion
Source: Proc Natl Acad Sci U S A. 2024 Apr 3;121(15):e2320505121. doi: 10.1073/pnas.2320505121 (PMC11009659; doi:10.1073/pnas.2320505121)
Supplement: Supplementary file 1 — Appendix 01 (PDF) [file pnas.2320505121.sapp.pdf]

PNAS

Supporting Information for

Complexin has a dual synaptic function as check-point protein in vesicle priming and as a promoter of vesicle fusion.

Francisco José López-Murcia[1], Kun-Han Lin[2], Manon M. M. Berns[1], Mrinalini Ranjan[1,3], Noa Lipstein[1], Erwin Neher[2,4*], Nils Brose[1,4], Kerstin Reim[1], Holger Taschenberger[1,*]

[1] Department of Molecular Neurobiology, Max Planck Institute for Multidisciplinary Sciences, 37075 Göttingen, Germany.

[2] Laboratory of Membrane Biophysics, Max Planck Institute for Multidisciplinary Sciences, 37077 Göttingen, Germany

[3] Göttingen Graduate School for Neurosciences, Biophysics, and Molecular Biosciences, Georg August University Göttingen, 37077 Göttingen, Germany

[4] Cluster of Excellence 'Multiscale Bioimaging' (MBExC), Georg August University Göttingen, 37073 Göttingen, Germany

* Correspondence: Erwin Neher, Holger Taschenberger

Email: eneher@mpinat.mpg.de, taschenberger@mpinat.mpg.de

This PDF file includes:

Supporting text
Figures S1 to S7
Table S1
SI References

Material and Methods

Mouse lines and animal maintenance

Mouse lines [C57/Bl6-Cplx1, (1), C57/Bl6-Syt7 (2)] were maintained in accordance with European Union Directive 63/2010/EU and ETS 123 (individually ventilated cages, specific pathogen-free conditions, 21 ± 1°C, 55% relative humidity, 12 h/12 h light/dark cycle). Mice received food and tap water *ad libitum* and were provided with bedding and nesting material. Cages were changed once a week. Animal health was controlled daily by caretakers and by a veterinarian. Health monitoring (serological analyses; microbiological, parasitological, and pathological examinations) was done quarterly according to FELASA recommendations with either NMRI sentinel mice or animals from the colony.

Mutant Syt7 C_2AB KI/KO mice containing point mutations in exons 11 and 13, and a Neo-resistance gene cassette flanked by LoxP sites were used in this study. In the absence of Cre recombination, expression of Syt7 was undetectable in mice that were homozygous for the C_2AB mutation. Cplx1 single knockout (ko) and Cplx1/Syt7 double knockout (dko) mouse pups of either sex originating from heterozygous crossing were identified by PCR genotyping (1, 2).

Slice preparation

Brainstem slices were prepared as described previously (3). P15–21 wt, $Cplx^{-/-}$ and $Cplx1^{-/-}Syt7^{-/-}$ littermate mice of either sex were used. Mice were decapitated and the brain was quickly removed and immersed into ice-cold low-Ca^{2+} artificial CSF (aCSF) containing the following (in mM): 125 NaCl, 2.5 KCl, 3 $MgCl_2$, 0.1 $CaCl_2$, 25 glucose, 25 $NaHCO_3$, 1.25 NaH_2PO_4, 0.4 ascorbic acid, 3 myo-inositol, and 2 Na-pyruvate at pH 7.3 when bubbled with carbogen (95% O_2, 5% CO_2). The brainstem was dissected out and glued onto the stage of a VT1000S vibratome (Leica). 200 µm-thick coronal slices containing MNTB and LSO were collected and incubated for 40 min at 35°C in normal aCSF and kept at room temperature (21–24°C) for up to 5 h thereafter. The composition of normal aCSF was identical to that of low-Ca^{2+} aCSF except that 2 mM $CaCl_2$ and 1 mM $MgCl_2$ were used.

Cerebellar slices were prepared as described in Chen et al. (4). P20–28 wt, $Cplx^{-/-}$ and $Cplx1^{-/-}Syt7^{-/-}$ littermate mice of either sex were used. Mice were decapitated and the whole brain was quickly removed and immersed into ice-cold low-Ca^{2+} solution containing the following (in mM): 87 NaCl, 2.5 KCl, 7 $MgCl_2$, 0.5 $CaCl_2$, 10 glucose, 25 $NaHCO_3$, 1.25 NaH_2PO_4, 75 sucrose at pH 7.3 when bubbled with carbogen (95% O_2, 5% CO_2). Parasagittal 290 µm-thick cerebellar slices from the vermis regions were collected and incubated for 40 min at 35°C in normal aCSF and kept at room temperature (21–24°C) for up to 5 h thereafter.

Electrophysiological recordings in acute slices

Patch-clamp recordings were made using an EPC-10 amplifier controlled by “Pulse” or “Patch-Master” software (HEKA Elektronik). Sampling intervals and low-pass filter settings were 20 μs and 5 kHz, respectively. Cells were visualized by using infrared light and differential interference contrast (DIC) microscopy or oblique illumination (Dodt gradient contrast) through 40× or 60× Olympus water-immersion objectives using upright microscopes (BX51WI, Olympus or Axioskop FS, Zeiss). During experiments, slices were continuously perfused with normal aCSF solution at room temperature.

Postsynaptic recordings were obtained from MNTB principal neurons (PNs), LSO PNs or Purkinje cells (PCs). Patch pipettes were pulled from borosilicate glass capillaries with filament (Science Products) on a P-97 Flaming/Brown Puller (Sutter Instruments) to have an open-tip resistance of 3–5 MΩ when filled with internal solution.

IPSCs were recorded in LSO PNs or PCs with an internal solution containing (in mM): 136 KCl, 15 K-gluconate, 10 EGTA, 2 $MgCl_2$, 2 Na_2ATP and 10 HEPES, pH 7.3 with KOH. EPSC were recorded in MNTB PNs with an internal solution containing (in mM): 140 Cs-gluconate, 20 TEA-Cl, 10 HEPES, 5 EGTA, 5 Na2-phosphocreatine, 5 ATP-Mg, 0.3 GTP, pH 7.3 with CsOH. AP-evoked EPSCs were recorded in the continuous presence of 1 mM kynurenic acid (kyn) in the bath solution to attenuate possible postsynaptic AMPAR saturation and desensitization (5-7). In the presence of 1 mM kyn, quantal size is reduced by ~87% and spontaneous and delayed quantal release events cannot be reliably resolved. Therefore, kyn was omitted from the bath when recording spontaneous and delayed asynchronous calyx EPSCs.

Patch pipettes were coated with dental wax to reduce stray capacitance. The holding potential was −70 mV. Series resistance (R_s) ranged from 4–10 MΩ. R_s compensation was set to ≥70% (2 μs delay for MNTB recordings, 100 μs delay for LSO or PC recordings). R_s compensation was reduced to ≤50% when recording spontaneous and delayed asynchronous calyx EPSCs.

Presynaptic recordings in cerebellar MLIs were performed in the current-clamp configuration with patch pipettes with an open-tip resistance of 5–8 MΩ when filled with internal solution containing (in mM): 125 K-gluconate, 20 KCl, 0.1 EGTA, 2 $MgCl_2$, 2 Na_2ATP, 0.4 GTP, 10 phosphocreatine and 10 HEPES, pH 7.3 with KOH. The holding current was set to maintain a resting membrane potential of approximately −65 mV. To elicit APs in presynaptic MLIs, depolarizing current pulses (400 pA, 4 ms duration) were injected. Glutamatergic EPSCs were elicited at calyx of Held synapses via a bipolar stimulating electrode placed halfway between the brainstem midline and the MNTB. Stimulation pulses of 100 μs duration were applied using a stimulus isolator unit (AMPI, Israel), with the output voltage set to 1–2 V above the threshold for AP generation (≤20 V). Glycinergic IPSCs were elicited in LSO PNs by afferent fiber stimulation using a bipolar

stimulation electrode placed above the ipsilateral MNTB and brief stimulus pulses (100 µs ~25 V) were applied.

Glycinergic and GABAergic IPSCs were pharmacologically isolated by application of 2 µM NBQX. Glutamatergic EPSCs was pharmacologically isolated by application of 5 µM strychnine. Mean AP-evoked IPSC and EPSC waveforms were obtained by averaging ≥5 consecutive sweeps recorded at a 15 s inter-sweep interval. To quantify delayed quantal release, up to 100 consecutive sweeps were collected at 15 s inter-sweep interval. Postsynaptic recordings with a leak current >300 pA were excluded from the analysis.

Immunostaining

Mice were anesthetized and perfused at room temperature in 4% paraformaldehyde (PFA) PB 0.1 M complying with national animal care guidelines (LAVES). Thereafter, dissected brains from Cplx1$^{-/-}$, Syt7$^{-/-}$ and littermate wt mice were immersed overnight at 4°C in 4% PFA 0.1 M PB. Forty micrometer-thick coronal sections including the brainstem Superior Olivary Complex (SOC) or parasagittal sections of the cerebellar cortex were cut at below 0°C and washed three times in PB 0.1 M. Floating sections were incubated for 1 h in blocking solution (5% normal goat serum, 0.5% Triton X-100 in 0.1 M PB) at room temperature. Primary antibodies (Abs) were dissolved in incubation buffer (3% normal goat serum, 0.3% Triton X-100 in 0.1 M PB) and applied overnight at 4°C while gently stirring. After washing three times for 10 min each time in 0.1 M PB, the tissue was incubated with secondary Abs in incubation buffer in the dark for 1.5 h at room temperature. Thereafter, sections were washed three times for 10 min each time in 0.1 M PB, mounted onto the glass microscope slides using Aqua-Poly/Mount (# 18606, Polysciences) and covered with glass coverslips. The following Abs were used: rabbit anti-Cplx1/2 (1:500; #122 002, Synaptic System), guinea pig anti-vesicular glutamate transporter 1 (VGLUT1, 1:500; #135 304, Synaptic System), guinea pig anti-vesicular GABA/Gly transporter (VGAT, 1:500; #131 004, Synaptic System), rabbit anti-Syt7 (1:500; #105 172, Synaptic System), and secondary goat-anti-rabbit AlexaFluor 488 (#A11008, ThermoFisher) and goat-anti-guinea pig AlexaFluor 555 (#A21435, ThermoFisher).

Offline analysis

Off-line analysis of electrophysiological data was performed in Igor Pro (Wavemetrics). Evoked EPSCs recorded in MNTB PNs were corrected for the remaining series resistance (R_s) using the R_s values stored in the data files and by assuming a linear I-V relationship with a reversal potential of +10 mV. IPSC and EPSCs were offset corrected and low-pass filtered with a cut-off frequency f_c = 3 and 5 kHz for IPSCs and EPSCs, respectively, using a digital Gaussian filter. Spontaneous quantal events occurring without previous conditioning stimulation were detected using a sliding template algorithm (8). Delayed quantal events following conditional stimulation were detected using a first derivative-based detection algorithm. Reliable event detection required

additional low-pass filtering of the first derivative with a cut-off frequency $f_c \leq 800$ Hz. Detection criteria were adapted for each recording in order to minimize false positive and false negative detection rates. Differential equations were solved numerically using the fifth-order Runge-Kutta-Fehlberg algorithm implemented in Igor Pro.

Decomposition of quantal release into distinct components using non-negative tensor factorization

Non-negative tensor factorization (NTF) of calyx of Held EPSC trains was performed similarly as previously described (9): EPSC trains evoked by afferent-fiber stimulation using trains consisting of 40 stimuli delivered at stimulation frequencies (f_{stim}) of 0.5, 1, 2, 5, 10, 20, 50, 100 and 200 Hz were recorded. For NTF, however, only data within the frequency range 5–200 Hz were used. To convert EPSC peaks into quantal content (m), we assumed an effective quantal size $q^* = -6.6$ pA in the presence of 1 mM kyn in the bath. The quantity q^* is slightly smaller than the mean mEPSC size to compensate for the temporal dispersion of quanta during AP-evoked synchronous release when deriving quantal content estimates from EPSC peak amplitudes instead of deconvolution (6, 9, 10).

At least three eEPSC train repetitions were analyzed for each stimulus frequency and the respective m_j values for each stimulus j were averaged. NTF data were standard tensors consisting of one layer, except for the two highest f_{stim} of 100 and 200 Hz for which two additional tensor layers were obtained by pre-conditioning the synapses with either 2 or 4 stimuli delivered at a frequency of 10 Hz. During data acquisition, synapses were allowed to rest after each stimulus train to completely recover from activity-induced changes in synaptic transmission back to resting conditions (15 s following ≤10 Hz trains, 25 s following 20 and 50 Hz, 40 s following 100 and 200 Hz). The order of the stimulus trains was pseudo-randomized for each synapse to avoid a contamination of the data with systematic run-down or run-up trends.

NTF analysis was performed in two sequential steps: (1) First, a two-component NTF was performed to obtain the two basefunctions BF_{TS} and $BF_{LS,RS}$ which represent release contributed by those SVs that were already in a tightly docked state (TS SVs) prior to stimulation and of those that were not (LS and RS SVs), respectively. Both BFs were initialized as previously described (9) and 200 iterations were used during which the goodness of fit was tracked. (2) Subsequently, a three-component NTF decomposition was performed providing three basefunctions BF_{TS}, BF_{LS} and BF_{RS} representing release of those SVs that were either in a tightly (TS SVs) or a loosely (LS SVs) docked state prior to stimulation and those SVs which were in neither of these two states (RS SVs), respectively. During this second step of analysis, the timecourse of BF_{TS} was constrained to the result of the previous two-component NTF decomposition, and only 100 iterations were used. The estimate for the mean M_{LS} was biased during iterations by shifting it at each cycle by 25–35% towards a pre-determined target value. This is necessary, because three-component NTF analysis does not ensure a unique separation between M_{LS} and M_{RS} (9). The

target value was calculated as described previously (10). In brief, our previous work (10) showed that the sum of TS and LS SVs at rest corresponds approximately to the total number of fast releasing SVs (*FRP*; Fig. 2A,B) in calyces (11, 12). We estimated *FRP* size from the cumulative quantal release during high-frequency stimulation after correction for pool replenishment (Fig. 2A) and subsequent extrapolation to infinite f_{stim} (Fig. 2B) (10, 13). We then subtracted the mean M_{TS} from the *FRP* to obtain an estimate for M_{LS}, which – provided all preexisting LS SVs are consumed during the trains – equals the number of LS SVs at rest. *FRP* estimates were obtained from cumulative release during high-frequency trains which, however, only incompletely deplete the *FRP* (14). We, therefore, obtained three individual *FRP* estimates (*FRP'*) for f_{stim} = 50, 100 and 200 Hz (Fig. 2A) and plotted 1 / *FRP'* vs. ISI (Fig. 2B). By extrapolating a regression line of this plot to ISI = 0 ms, we effectively obtain an *FRP* estimate for infinite f_{stim} and assume that this approach compensates for incomplete *FRP* depletion (10, 13).

Statistical analysis

Original data are presented as mean ±SEM. Unless otherwise stated, statistical significance of differences in mean was evaluated using permutation tests when comparing two groups and Wilcoxon rank sum test with Bonferroni adjustment when comparing three groups. $p < 0.05$ was taken as the level of statistical significance.

Kinetic scheme for SV priming and fusion

Timecourses of AP-evoked synchronous release during stimulus trains (Fig. 2C) were simulated using a kinetic scheme (Fig. S3A) as previously described (10). This scheme implies that (1) docking/priming of SVs occurs at a single type and a fixed number of release sites, (2) SV docking/priming steps are reversible and vesicle states are in dynamic equilibrium with each other at rest and during inter-stimulus intervals, (3) only SVs equipped with a mature release machinery (TS and TSL SVs, see below) are fusion-competent, and (4) the docking/priming rate constants for the ES→LS transition (k_1) and for the LS→TS transition (k_2) are Ca^{2+}-dependent, while all undocking/unpriming rate constants have fixed values.

We assume a constant number of functionally identical release sites (N_{tot}), which at a given time *t* can either be empty and available for docking/priming (N_{ES}), occupied with a docked/primed SV, or empty and unavailable for docking/priming (N_{ERS}), i.e. the site can be 'refractory' following a release event. Docking and priming of a SVs proceeds by transition through two sequential maturation states: SVs are initially loosely docked (LS SVs) before they mature into a tightly docked fusion competent SV (TS SVs). In addition, we introduced a fusion-competent SV state, which is similar to state TS but labile (TSL). A fraction (κ) of LS SVs instantaneously transitions into this state (TSL SVs) immediately after each AP. TSL SVs rapidly return to state LS due to a relatively high backward rate constant for the LS←TSL transition (b_3). Following SV fusion events, vacated release sites instantaneously turn into empty and refractory sites (N_{ERS}) before

becoming available for refilling (N_{ES}). Thus, the entire pool of docked/primed SVs at a given time t can be subdivided into LS, TS and TSL subpools (SP_{LS}, SP_{TS}, SP_{TSL}, respectively), such that

$$N_{tot} = N_{ES}(t) + SP_{LS}(t) + SP_{TS}(t) + SP_{TSL}(t) + N_{ERS}(t) \quad (1)$$

The following coupled differential equations describe temporal changes in state occupancies at rest and during inter-stimulus intervals:

$$\frac{d}{dt}N_{ES}(t) = -k_1 \cdot N_{ES}(t) + b_1 \cdot SP_{LS}(t) + b_4 \cdot N_{ERS} \quad (2)$$

$$\frac{d}{dt}SP_{LS}(t) = -(b_1 + k_2) \cdot SP_{LS}(t) + b_2 \cdot SP_{TS}(t) + b_3 \cdot SP_{TSL}(t) + k_1 \cdot N_{ES}(t) \quad (3)$$

$$\frac{d}{dt}SP_{TS}(t) = -b_2 \cdot SP_{TS}(t) + k_2 \cdot SP_{LS}(t) \quad (4)$$

$$\frac{d}{dt}SP_{TSL}(t) = -b_3 \cdot SP_{TSL}(t) \quad (5)$$

$$\frac{d}{dt}N_{ERS}(t) = -b_4 \cdot N_{ERS}(t) \quad (6)$$

The backward rate constants b_1, b_2, b_3 and b_4 have fixed values, and the two forward rate constants k_1 and k_2 are modeled as Ca^{2+}-dependent quantities which increase linearly with the 'effective' $[Ca^{2+}]_i$ according to:

$$k_1(t) = k_{1,rest} + \sigma_1 \cdot ([Ca^{2+}](t) - [Ca^{2+}]_{rest}) \quad (7)$$

$$k_2(t) = k_{2,rest} + \sigma_2 \cdot ([Ca^{2+}](t) - [Ca^{2+}]_{rest}). \quad (8)$$

$[Ca^{2+}]_{rest}$ represents the resting $[Ca^{2+}]_i$, which was assumed to be 50 nM (15). σ_1 and σ_2 are linear slope factors characterizing the Ca^{2+} dependence of the SV priming steps. For each release event, the quantal content m_j of the $EPSC_j$ triggered by stimulus j was calculated as the product of $p_{fusion,j} \cdot (SP_{TS}(t_j) + SP_{TSL}(t_j))$ with all quantities evaluated immediately before stimulus arrival. Index j indicates stimulus index, j = 1 to 40. SP_{TS} and SP_{TSL} were decremented by their contribution to m_j and N_{ERS} was incremented by m_j. $[Ca^{2+}](t)$ was assumed to decay back to its resting value $[Ca^{2+}]_{rest}$ with a double-exponential timecourse

$$[Ca^{2+}](t) = \Delta[Ca^{2+}]' \cdot f_{fast} \cdot \exp\left(\frac{-(t - t_j)}{\tau_{fast}}\right) \quad (9)$$

$$+\Delta[Ca^{2+}]' \cdot (1 - f_{fast}) \cdot \exp\left(\frac{-(t - t_j)}{\tau_{slow}}\right) + [Ca^{2+}]_{rest}$$

where Δ[Ca^{2+}]' is the amplitude of the AP-evoked 'effective' Ca^{2+} transient (Δ[Ca^{2+}]) corrected for dynamic changes in presynaptic Ca^{2+} influx as previously described (10). f_{fast}, τ_{fast} and τ_{slow} denote fraction and decay time constant of the fast decay component and decay time constant of the slow decay component of the Ca^{2+} transient, respectively, and t_j is the onset time of the jth stimulus.

In resting synapses, N_{ERS} and SP_{TSL} eventually approach zero, such that Equations 1–4 provide the following analytical expressions for resting occupancies at t = 0:

$$N_{ES,rest} = N_{tot} \cdot b_1 \cdot b_2 / (b_1 \cdot b_2 + b_2 \cdot k_{1,rest} + k_{1,rest} \cdot k_{2,rest}) \quad (10)$$

$$SP_{LS,rest} = N_{tot} \cdot b_2 \cdot k_{1,rest} / (b_1 \cdot b_2 + b_2 \cdot k_{1,rest} + k_{1,rest} \cdot k_{2,rest}) \quad (11)$$

$$SP_{TS,rest} = N_{tot} \cdot k_{1,rest} \cdot k_{2,rest} / (b_1 \cdot b_2 + b_2 \cdot k_{1,rest} + k_{1,rest} \cdot k_{2,rest}) \quad (12)$$

$$SP_{TSL,rest} = N_{ERS,rest} = 0 \quad (13)$$

To simulate the time-resolved rate of spontaneous and delayed release ($\xi(t)$) in wt and ko synapses simultaneously with discrete AP-evoked synchronous release events (Fig. 5) or at rest (Fig. 6), the kinetic scheme described above was extended in two ways (Fig. S3B): (*i*) A state transition from ES into a fusion-competent 'faulty' SV state was added as a parallel branch to the ES↔LS↔TS priming pathway. Thus, FS SVs effectively compete with LS, TS and TSL SVs for the fixed number of release sites (N_{tot}). Forward (k_f) and backward (b_f) rate constants of the reversible ES↔FS transition were allowed to differ between wt and ko synapses, and k_f was assumed to be linearly dependent on Ca^{2+} analogous to k_1 and k_2 (Equations 7 and 8). (*ii*) In addition to the instantaneous release of m_j quanta at stimulus j, spontaneous release and delayed release were calculated before, during and after stimulation as the products ($SP_{TS}(t)$ + $SP_{TSL}(t)$) · $\gamma_t(t)$ and $SP_{FS}(t) \cdot \gamma_f(t)$, where $\gamma_t(t)$ and $\gamma_f(t)$ are the fusion rate constants of tightly docked and 'faulty' SVs at [Ca^{2+}](t) (Equation 9) according to the simulated [Ca^{2+}]-fusion relationships (Fig. 5C) and the simulated timecourse of the effective [Ca^{2+}]$_i$ (Equation 9, Fig. 5B).

The relationship between the fusion rate constants γ_t and [Ca^{2+}]$_i$ was modeled for wt and ko synapses (Fig. 5C2) using the allosteric model for SV fusion (15). The model was solved numerically for step-like [Ca^{2+}]$_i$ elevations to various concentrations with a duration of 45 ms and assuming a $SP_{TS,rest}$ as obtained by NTF decomposition. Values of the parameters of the

allosteric model were adjusted for wt and ko synapses separately. Starting guess values for parameter values for wt mice were chosen according to Kochubey and Schneggenburger (16). The value of the intrinsic release willingness of TS SVs, l_+, was subsequently adjusted to approximate the experimentally observed sEPSC frequency. Additionally, the value of *f* (15) was adjusted so that experimentally obtained AP-evoked EPSC amplitudes could be simulated (3, 17). Simulations of EPSCs were performed by driving the allosteric model with a Ca^{2+} transient describing the microdomain $[Ca^{2+}]_i$ in mature calyx synapses (17). Subsequently, the resulting release rates were convolved with an averaged mEPSC waveform. The same procedure was repeated for the TS SVs in the ko condition.

Parameters were tuned so that simulated AP-evoked EPSC amplitudes and sEPSC frequencies matched experimentally observed values. The values for *f* and l_+ were altered such that the maximum obtainable release rate was equal in wt and ko synapses, consistent with the experimental finding that increasing esternal $[Ca^{2+}]$ leads to a rescue of the ko phenotype (1). Furthermore, the Ca^{2+}-binding affinity was altered to create a rightward shift in the $[Ca^{2+}]$-transmitter release relationship (Fig. 5C2) consistent with previously reported data (1, 18). The relationship between $[Ca^{2+}]_i$ and fusion rate for the tightly docked SVs was obtained once and used as a lookup table when numerically solving the model. This approach does not take into account the time required for equilibrating the Ca^{2+}binding sites. However, as binding and unbinding of Ca^{2+} to the vesicular Ca^{2+} sensor occur at a much faster time scale compared to the other reactions in this model, the effect of this simplification on the predicted spontaneous and asynchronous release rate is negligible.
It is important to note that (*i*) the 'effective' $[Ca^{2+}]_i$ driving spontaneous and delayed release did not exceed 1 µM during the simulations and (*ii*) AP-evoked synchronous EPSCs were modeled as instantaneous release events. Thus, only the $[Ca^{2+}]$ range between 50 nM and 1 µM of the simulated $[Ca^{2+}]$-fusion relationships is relevant during simulations. The extended kinetic model as described above was solved for steady-state at the beginning of the simulation and then allowed to evolve by numerical integration.

Approximating p_{fusion} from 5 to 20 Hz EPSC trains

For inter-stimulus intervals (Δt_{ISI}) of 200 to 50 ms, corresponding to f_{stim} = 5–20 Hz, the terms $\Delta t_{ISI} \cdot k_{1,rest}$ and $\Delta t_{ISI} \cdot k_{2,rest}$ are smaller by an order of magnitude than the amount of AP-induced SV priming given by the integrals $\int_t^{t+\Delta t_{ISI}} \sigma_1 \cdot ([Ca^{2+}](t) - [Ca^{2+}]_{rest}) \cdot dt$ and $\int_t^{t+\Delta t_{ISI}} \sigma_2 \cdot ([Ca^{2+}](t) - [Ca^{2+}]_{rest}) \cdot dt$. In addition, $\Delta t_{ISI} \cdot b_1$ and $\Delta t_{ISI} \cdot b_2$ are much smaller than the amounts of AP-induced ES→LS and LS→TS transitions. Thus, SVs priming proceeds predominantly from left to right in the scheme of Fig. S3A. Neglecting the small terms, one arrives at a simple equation for approximating the initial value of p_{fusion} ($p_{fusion,1}$) from paired-pulse ratio

and relative steady-state depression: $p_{fusion,1} = (1 - \frac{PPR}{r_p})/(1 - \frac{D_m}{D_{LS}})$, where $D_m = m_{ss}/m_1$ and $D_{LS} = \overline{SP}_{LS,ss}/SP_{LS,1}$ (10). Here, the parameter $r_p = p_{fusion,2}/p_{fusion,1}$ was introduced, which is very close to 1 for 5–20 Hz stimulation. Considering that only little reduction in the size of SP_{LS} is expected during 5–20 Hz EPSC trains, an approximate expression for $p_{fusion,1}$, assuming $r_p \approx 1$ and $D_{LS} \approx 1$, is simply: $p_{fusion,1} \approx (1 - PPR)/(1 - D_m) = (1 - EPSC_2/EPSC_1)/(1 - EPSC_{ss}/EPSC_1)$ (10).

For 5–20 Hz EPSC trains in wt synapses, PPR was between 0.64 and 0.59 and D_m was between 0.32 and 0.22, corresponding to $p_{fusion,1} \approx 0.52$. For Cplx1 ko synapses, the respective values were $PPR =$ 0.64 to 0.59 and D_m = 0.32 to 0.22, corresponding to $p_{fusion,1} \approx 0.33$.

Western Blot Analysis

Crude synaptosomal fractions (P2) were obtained from cortical brain tissue of P15–16 Cplx1 ko, Syt7 ko and Cplx1/Syt7 dko mice, and wt littermates as previously described (13). Between 2 to 20 µg protein were separated on 4%–12% gradient Bis-Tris polyacrylamide gels (Invitrogen) and blotted onto nitrocellulose membranes. Equal protein load per lane (mutant vs. control) was further verified by a reversible membrane staining protocol (MemCode; Pierce). The following antibodies were used: mouse monoclonal (mm)-anti-Synaptotagmin 1 (SySy 105 001), rabbit polyclonal (rp)-anti-Synaptotagmin-2 (SySy, 105 223), rp-anti-Synaptotagmin-7 (SySy 105 172) and rp-anti-Complexin-1/2 (SySy, 122 002). After incubation with corresponding secondary antibodies (peroxidase-AffiniPure goat anti-mouse IgG or peroxidase-AffiniPure goat anti-rabbit IgG antibody), Western blot signals were detected by enhanced chemiluminescence (Cytiva) using an INTAS imager (INTAS Science Imaging).

Confocal microscopy and image analysis

Images were acquired with a Leica SP2 confocal microscope (Leica Microsystems) with 488 nm (Ar) and 561 nm (He-Ne) lasers for excitation at high magnification (40× oil-immersion objective, NA 1.25) using a resolution of 1024 × 1024 pixels. Three-dimensional reconstruction at high magnification was obtained by collecting z-axis stacks with horizontal image planes separated by ~0.5 µm resulting in ≥ 1 cross-sections trough individual synaptic boutons. For low magnification, step sizes of ≥ 1 µm were used. Image analysis was carried out using ImageJ (NIH). Calyx of Held terminals were identified as vGluT1 immunopositive ring-like structures surrounding MTNB PNs. Putative glycinergic and GABAergic presynaptic boutons were identified in maximum intensity projections of anti-VIAAT fluorescence image stacks in the LSO region and in the cerebellar PC layer. A particle-analysis algorithm implemented in ImageJ was used to identify presynaptic boutons (bouton size restricted to 0.5–2 µm^2) and each identified punctum was stored as a region of interest (ROI) and used to quantify intensities of anti-Cplx1/2 or anti-Syt7 immunofluorescence.

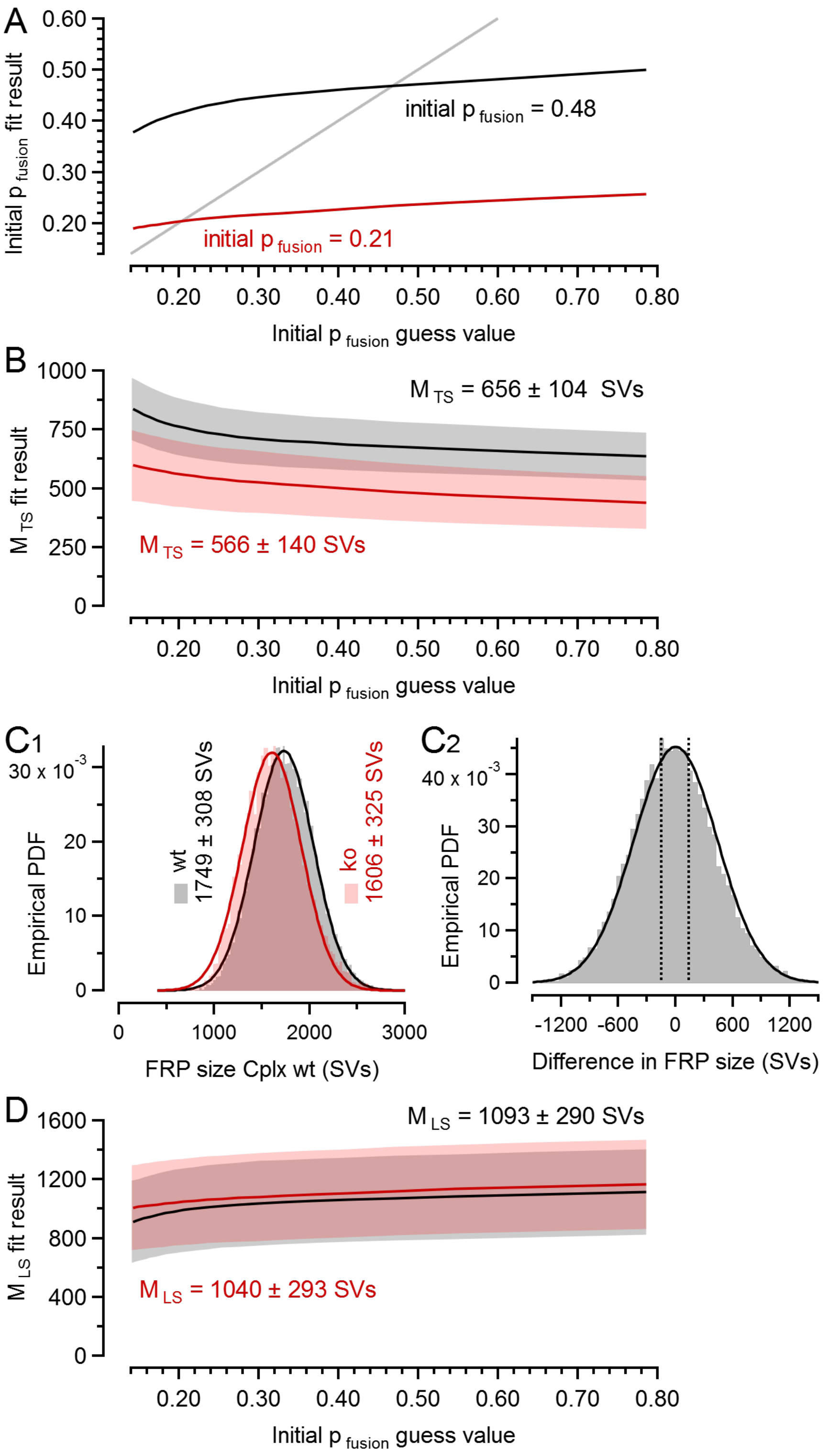

Fig. S1. Uncertainties associated with the estimates for initial p_{fusion} and the mean values of *FRP*, M_{TS} and M_{LS} across all wt and ko calyx of Held synapses tested.

A, NTF decomposition-derived estimates for initial p_{fusion} plotted as a function of the corresponding starting guess values for wt (black) and ko (red) calyx synapses. For an NTF

decomposition analysis, starting guess values are assigned to each of the relevant parameters, deviations between model predictions and data are calculated and minimized by an iterative procedure. Typically 200 iterations are performed during which parameters are varied by the algorithm and reach a final value at which χ^2 is minimized. The two curves show final values of initial p_{fusion}, plotted against the respective starting guess values for the two genotypes. For a 5.5fold change in guess values (from 0.14 to 0.78), the NTF fit results changed only 1.3fold for wt (*black*; from 0.38 to 0.50) or 1.7fold for ko (*red*; from 0.15 to 0.26). Importantly, the lowest p_{fusion} estimate for wt was about twofold larger than the highest p_{fusion} estimate for ko synapses, demonstrating a robust reduction of the NTF-based p_{fusion} estimate for Cplx1-lacking synapses. The p_{fusion} fit result that showed the smallest deviation from the corresponding starting guess value, as indicated by the crossover of the respective curve with the unit line (*gray*), was accepted as the final p_{fusion} estimate (values given next to the plots).
B, NTF decomposition-derived estimates for M_{TS} plotted as a function of the guess values for initial p_{fusion} for wt (*black*) and ko (*red*) calyx synapses. NTF decomposition provides an M_{TS} size estimate for each of the 14 wt and 13 ko synapses. These individual M_{TS} estimates were used to calculate ±SEM (shaded areas). The final M_{TS} estimate (values given next to the plots) were those obtained by the NTF decomposition which generated the final p_{fusion} value.
C, Bootstrap analysis of *FRP* estimates (**C1**) and differences in *FRP* size between wt and ko (**C2**). **C1**, An empirical probability density function (PDF) of *FRP* sample means was estimated by using a balanced bootstrap approach (every experimental observation appeared exactly the same number of times in the total population of 20,000 bootstrap resamples) for wt (*gray*) and ko (*light red*) synapses (19, 20). For each bootstrap resample, the *FRP* size was estimated by analyzing 50 Hz, 100 Hz and 200 Hz EPSC trains exactly as illustrated in Fig. 2A and B. Mean *FRP* values ± SEM (SD of the *FRP sizes* among bootstrap resamples) were 1749 ± 308 SVs for wt and 1606 ± 325 SVs for ko synapses. **C2**, An empirical PDF for the differences in *FRP* size between wt and ko synapses under the null hypothesis (H0) was estimated from the two populations of wt and ko bootstrap resamples after subtracting their respective means. The observed difference between wt and ko *FRP* estimates was 143 SVs. The two-tailed probability for observing differences ≥143 SVs (dotted lines) is 0.742. The solid lines in **C1** and **C2** represent Gaussian functions fitted to the PDFs.
D, NTF decomposition-derived estimates for M_{LS} plotted as a function of the guess values for initial p_{fusion} for wt (black line) and ko (red line) calyx synapses. For each synapse, M_{LS} was calculated as the difference $FRP - M_{TS}$. The final M_{LS} estimates (values given next to the plots) were obtained by using the final M_{TS} estimates as given in **B**. Assuming Gaussian error propagation, SEM estimates for M_{LS} (shaded areas) can be derived from the relationship $SEM_{FRP}{}^2 = SEM_{TS}{}^2 + SEM_{LS}{}^2 + 2 \cdot Cov(M_{TS}, M_{LS})$. Neglecting the correction for covariance, one obtains $SEM_{LS} = \sqrt{SEM_{FRP}{}^2 - SEM_{TS}{}^2}$, which therefore represents an upper bound.

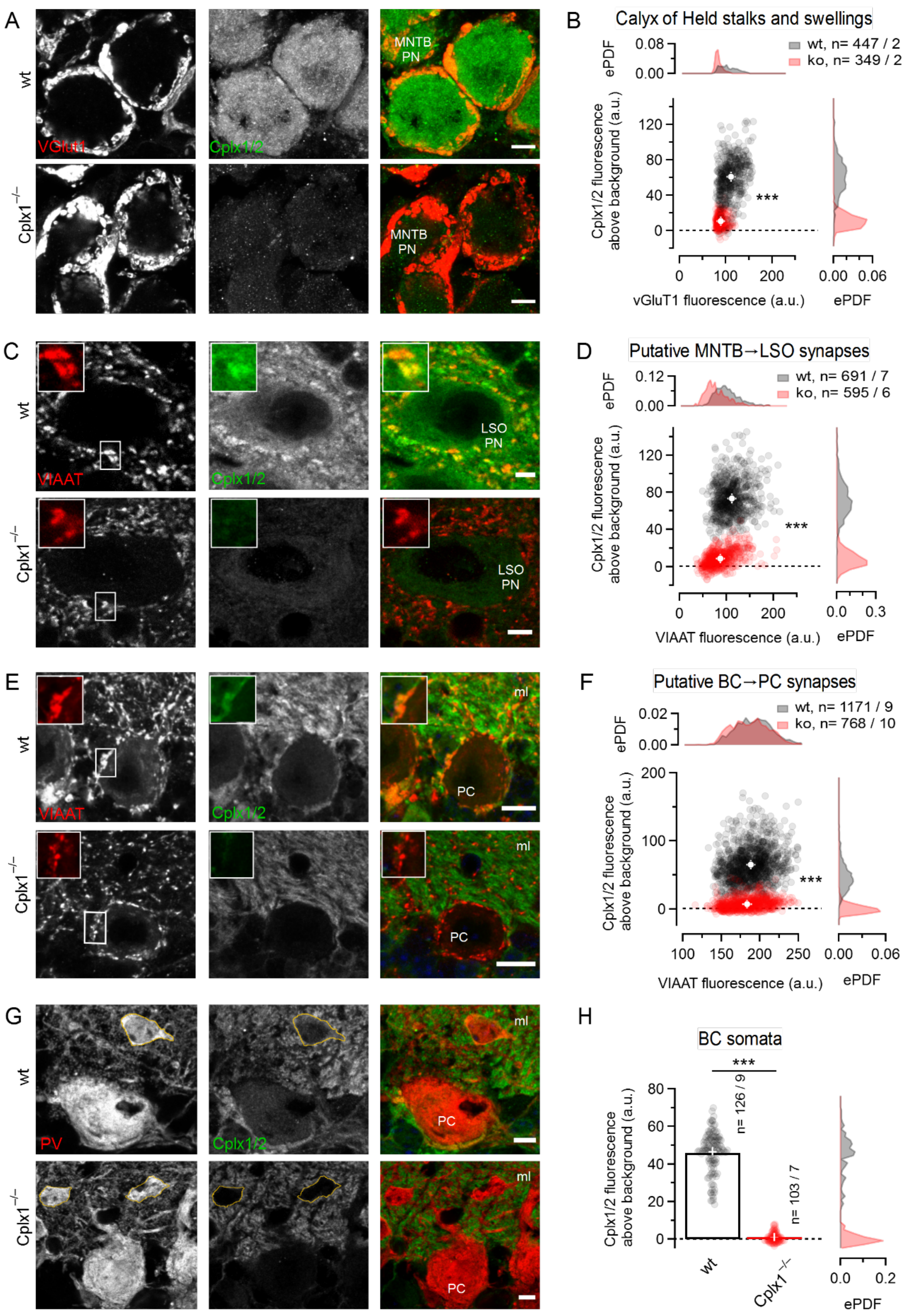

A
B
Calyx of Held stalks and swellings
wt, n= 447 / 2
ko, n= 349 / 2
ePDF
Cplx1/2 fluorescence above background (a.u.)
vGluT1 fluorescence (a.u.)
MNTB PN
VGluT1
Cplx1/2
wt
Cplx1−/−
***
C
D
Putative MNTB→LSO synapses
wt, n= 691 / 7
ko, n= 595 / 6
VIAAT
LSO PN
VIAAT fluorescence (a.u.)
E
F
Putative BC→PC synapses
wt, n= 1171 / 9
ko, n= 768 / 10
ml
PC
G
H
BC somata
PV
n= 126 / 9
n= 103 / 7

Fig. S2. Cplx1 is the major Cplx paralogue expressed presynaptically in P17-21 glutamatergic calyx of Held synapses, P15-19 glycinergic MNTB→LSO synapses and P20-28 GABAergic MLI→PC Synapses.

A,**B**, Quantification of presynaptic anti-Cplx1/2 fluorescence in calyces of brainstem sections obtained from wt (**A,** *top row*) and ko (**A,** *bottom row*) mice. Maximum-intensity projections were obtained from stacks of 2–3 confocal images of P15 MNTB. Presynaptic compartments were identified by anti-VGluT1 immunolabeling (*left column*, red color in *right column*). Sections were co-stained with anti-Cplx1/2 antibodies (*middle column*, green color in *right column*) to demonstrate presence or absence of Cplx1 in presynaptic calyces of wt (*top row*) and ko (*bottom row*) mice, respectively. A scatter plot of anti-Cplx1/2 versus anti-vGluT1 fluorescence intensity levels in calyx stalks and swellings surrounding MNTB PNs in wt (*gray*) and ko (*red*) mice is shown in **B**. Cplx1/2 fluorescence levels were background-corrected by subtracting the average fluorescence measured of nonspecific outside of the tissue regions. Empirical probability density functions (ePDFs, bin width 5 a.u.) for anti-vGluT1 fluorescence intensity and anti-Cplx1/2 fluorescence intensity are shown above and to the right of the scatter plot, respectively.
C,**D**, Similar analysis as shown in **A**,**B** but for MNTB→LSO synapses. Presynaptic glycinergic boutons decorating PNs of the LSO were identified by anti-VIAAT immunolabeling (*left column*, red color in *right column*). Anti-Cplx1/2 fluorescence (*middle column*, green in *right column*) intensity levels were strongly reduced in VIAAT$^+$ boutons surrounding LSO PNs in sections obtained from ko mice (**C,** *bottom row*) in comparison to those obtained from wt mice (**C,** *top row*), with little overlap between the two ePDFs (**D**, *right*). Insets show VIAAT$^+$ putative glycinergic boutons at higher magnification.
E,**F**, Similar analysis as shown in **A**,**B** but for MLI→PC synapses. Presynaptic boutons decorating PCs were identified by anti-VIAAT immunolabeling (*left column*, red color in *right column*). anti-Cplx1/2 fluorescence intensity levels were strongly reduced in VIAAT$^+$ boutons surrounding PC somata in sections obtained from ko mice (**E**, *bottom row*) in comparison to those obtained from wt mice (**E,** *top row*), with little overlap between the two ePDFs (**F**, *right*). Insets show VIAAT+ putative GABAergic boutons at higher magnification.
G,**H**, Quantification of anti-Cplx1/2 fluorescence intensity levels in Parvalbumin-positive (PV$^+$) putative MLIs in wt and ko mice. Maximum-intensity projections were obtained from image stacks of 2–3 confocal images of PV$^+$ (*left column*, red color in *right column*) putative GABAergic MLIs (outlined in yellow) adjacent to PV$^+$ PCs. Sections were co-stained with anti-Cplx1/2 antibodies (*middle column*, green color in *right column*) to demonstrate presence or absence of Cplx1 in MLI somata in wt (**G,** *top row*) and ko (**G,** *bottom row*) mice, respectively. In sections prepared from P22 Cplx1 ko mice (*bottom row*), anti-Cplx1,2 immunolabeling was not detected in MLI somata, indicating absence of Cplx2 (**H**).
MNTB PN = principal neuron of the MNTB, LSO PN = principal neuron of the LSO, ml = molecular layer, PC = Purkinje cell, scale bar = 5 µm. (*** $p<0.001$)

A

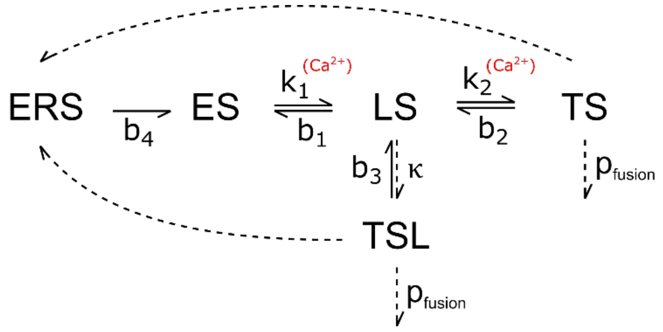

B

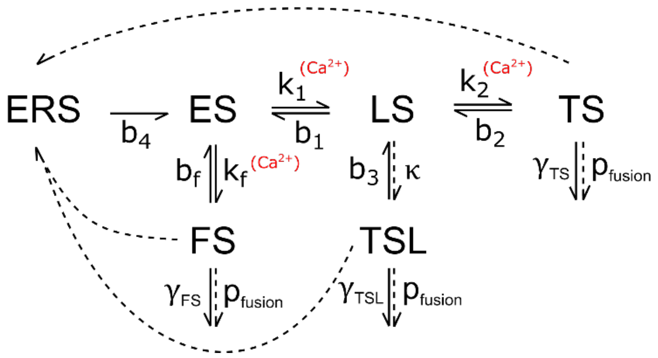

Fig. S3. Kinetic schemes for simulating AP-evoked synchronous, and spontaneous and delayed released before, during and after stimulus trains at calyx of Held synapses.

A, A five state model describing SV docking/priming and fusion was used to simulated AP-evoked synchronous release during stimulus trains in wt and ko calyx synapses. SVs dock at empty sites (ES) and subsequently transition from a loosely docked state (LS) to a tightly docked state (TS) of which only the latter is fusion-competent. Quantal contents of AP-triggered release were calculated as the product of the number of TS SV times p_{fusion} with both quantities evaluated at the time of AP arrival. Each AP shifts a fraction κ of LS SVs to a labile TS state (TSL). TSL SVs have the same p_{fusion} as TS SVs but rapidly transition back to the LS state by a rate constant b_3. Following fusion events, release sites are empty and unavailable for docking ('refractory'; ERS) and transition back to the ES by a time constant b_4 to become available for new SVs to dock. Forward (priming) rate constants k_1 and k_2 are Ca^{2+} dependent while backward (unpriming) rate constants b_1 and b_2, and also b_3 and b_4 are fixed.

B, To simulate spontaneous and delayed release in wt and ko calyx synapses, the kinetic scheme in **A** was extended by a 'faulty' state (FS). SV priming of the 'faulty' state may proceed sequentially analogous to the LS→TS transition. For simplicity, such consecutive states are lumped together here in a single 'faulty' SV state. The fusion rate $\xi(t)$ was calculated in a time-resolved manner as the product of the number of TS, TSL and FS SVs times the respective γ_{fusion} at $[Ca^{2+}](t)$ according to the simulated $[Ca^{2+}]_i$-fusion relationships (Fig. 5C) and the simulated timecourse of the effective $[Ca^{2+}]_i$ (Equation 9, Fig. 5B). All quantities were evaluated at each numerical integration step.

State transitions in **A** and **B** represented by dashed arrows indicate instantaneous transitions, while those represented by solid lines occur with rate constants as shown next to the arrows.

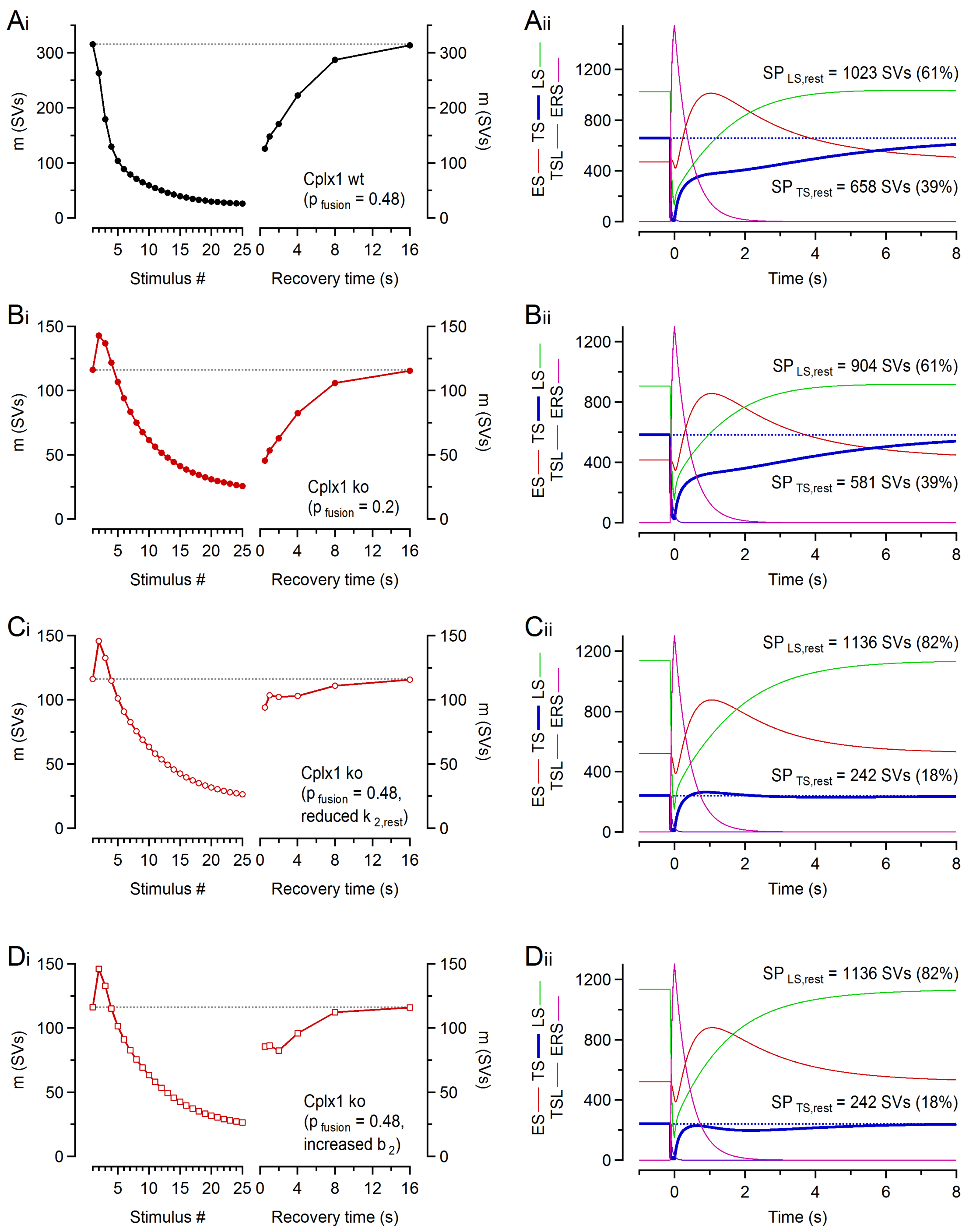

Fig. S4. Numerical simulations of recovery from synaptic depression induced by conditioning 200 Hz trains consisting of 25 stimuli.

A, Simulated timecourse of quantal contents (m) during the conditioning 200 Hz stimulation (*left*; m plotted versus stimulus number) and during recovery from synaptic depression (*right*; m plotted versus recovery time) in wt calyx synapses (**Ai**). Recovery was probed using a single stimulus delivered at an interval of 0.5, 1, 2, 4, 8 or 16 s following cessation of stimulation. The dotted line in **A** marks the initial synaptic strength. The timecourses of model state occupancies for the simulation shown in **Ai** are illustrated in **Aii**, with t = 0 s representing the onset of the recovery period following conditioning stimulation. Initial occupancies of the LS and TS states are given

next to the traces. The temporal dynamics of the TS SV subpool are indicated by the bold blue trace and its initial occupancy is marked by the dotted blue line.
B, Similar simulation as shown in **A** but for Cplx1 ko synapses.
Model parameters in **A** and **B** are identical to those given in Table S1. The simulated recovery timecourses in **A** and **B** match previously published experimental observations (3, 13), with full recovery from depression requiring ~16 s.
C, Similar simulation for Cplx1 ko synapses as shown in **B.** However, p_{fusion} was kept identical to wt synapses (0.48) and, alternatively, $k_{2,\text{rest}}$ was reduced by a factor of ~0.33, which results in a shift of the balance between LS and TS SVs at rest (10). This Cplx1 ko model reproduces initial synaptic strength and STP timecourse adequately (**Ci**, *left*) but fails to reproduce the slow recovery from synaptic depression (**Ci**, *right*), because the small TS subpool is quickly replenished from the large LS subpool during Ca^{2+}-enhanced priming after the conditioning 200 Hz stimulation (**Cii**).
D, Similar simulation for Cplx1 ko synapses as shown in **C.** However, instead of reducing $k_{2,\text{rest}}$, the value of b_2 was increased by a factor of ~3.02. Again, initial synaptic strength and STP timecourse of ko synapses were adequately reproduced (**Di**, *left*), but recovery from depression was too rapid (**Di**, *right*).
In conclusion, the Cplx1 ko model variants illustrated in **C** and **D** had to be rejected, because, in addition to contradicting NTF-based p_{fusion} estimates (Figs. 1E and S1A), they failed to reproduce experimentally observed time course of recovery from depression.

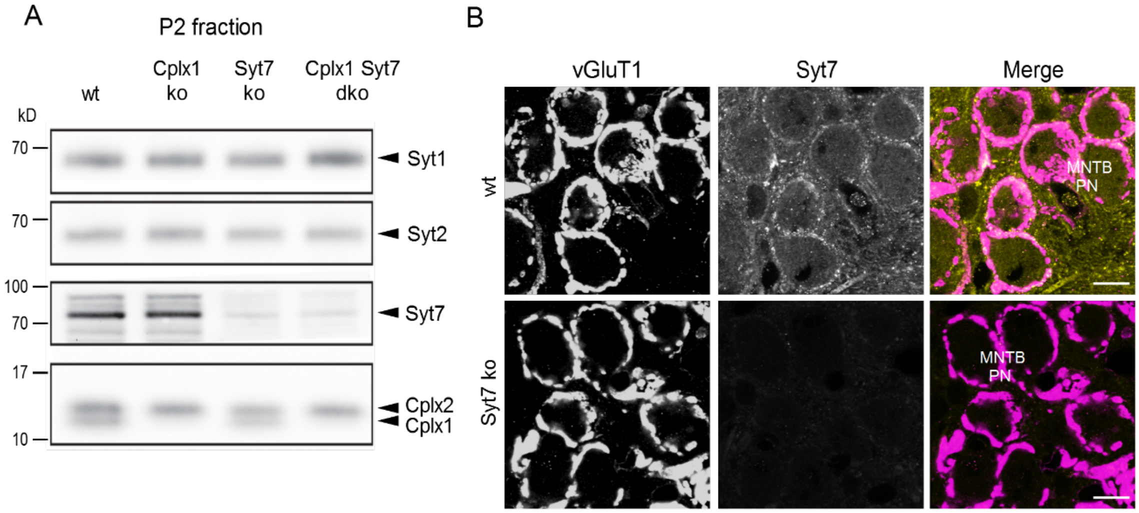

Fig. S5. Synaptotagmin 7 expression in calyx of Held terminals.
A, Western blot analysis of Syt1, Syt2, Syt7 and Cplx1,2 expression using crude P2 synaptosome fractions prepared from cortical brain tissue of wt, Cplx1 ko, Syt7 ko, and Cplx1,Syt7 dko mice. Note the similar expression levels of Syt1 and Syt2 in all four genotypes. As expected, Syt7 was undetected in brain tissue derived from Syt7 ko and Cplx1,Syt7 dko mice, and Cplx1 was undetected in brain tissue derived from Cplx1 ko and Cplx1,Syt7 dko mice.
B, Maximum-intensity projections acquired from confocal image stacks of MNTB PNs surrounded by presynaptic calyx of Held terminals immunolabeled for the presynaptic marker protein vGluT1 (*left column*, magenta in the *right column*) in sections obtained from a wt (*top row*) and a Syt7 ko (*bottom row*) mouse. Sections were co-stained with an anti- Syt7 antibody (*middle column*, yellow in the *right column*) to demonstrate presence and absence of Syt7 expression in calyx of Held terminals of wt and Syt7 ko mice, respectively. Scale bar, 10 µm.

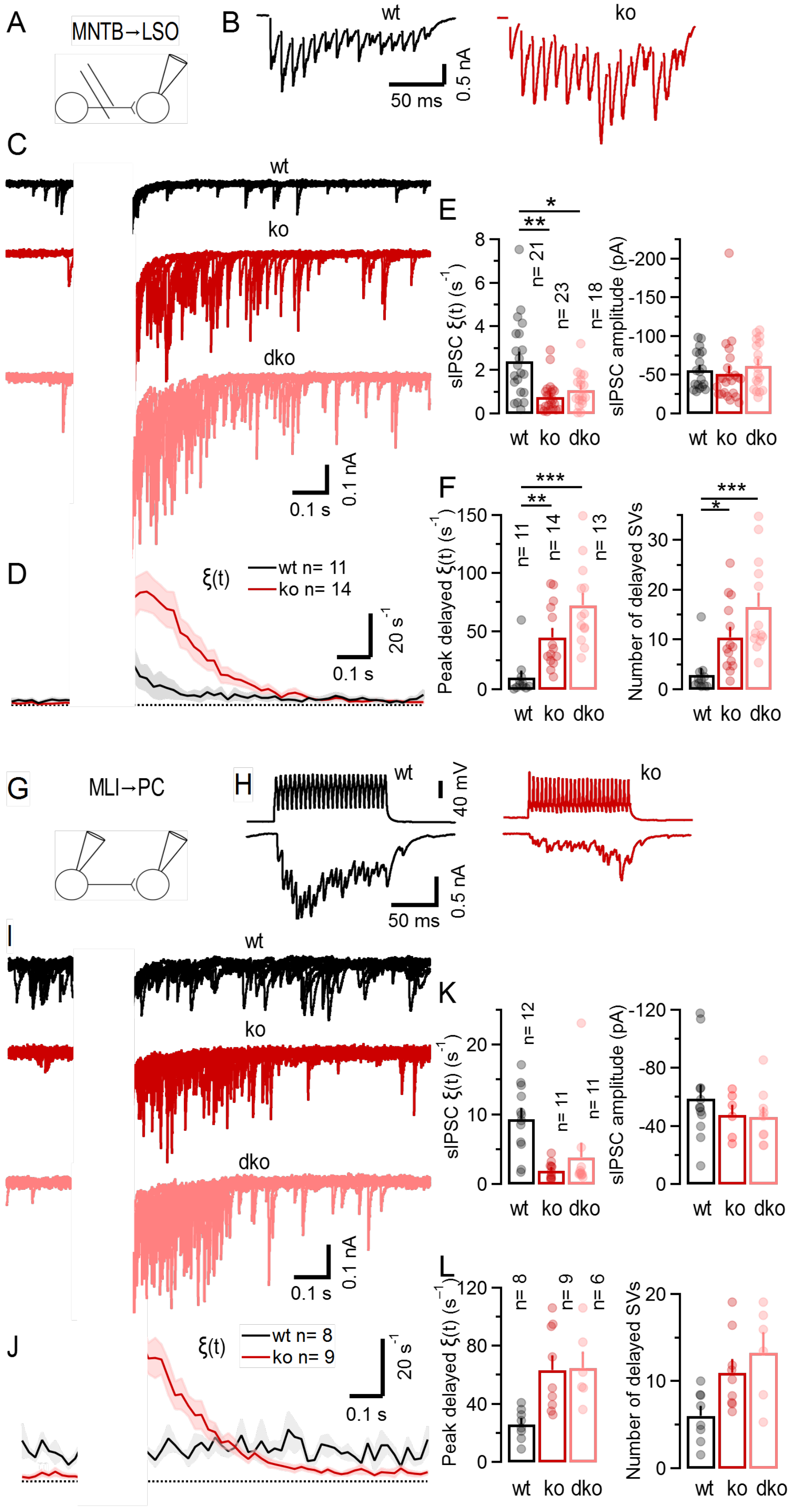
A
MNTB→LSO
B
wt
ko
50 ms
0.5 nA
C
wt
ko
dko
0.1 s
0.1 nA
D
ξ(t)
wt n= 11
ko n= 14
0.1 s
E
sIPSC ξ(t) (s⁻¹)
n= 21
n= 23
n= 18
sIPSC amplitude (pA)
wt ko dko
F
Peak delayed ξ(t) (s⁻¹)
n= 11
n= 14
n= 13
Number of delayed SVs
wt ko dko
G
MLI→PC
H
wt
ko
40 mV
50 ms
0.5 nA
I
wt
ko
dko
0.1 s
0.1 nA
J
ξ(t)
wt n= 8
ko n= 9
0.1 s
K
sIPSC ξ(t) (s⁻¹)
n= 12
n= 11
n= 11
sIPSC amplitude (pA)
wt ko dko
L
Peak delayed ξ(t) (s⁻¹)
n= 8
n= 9
n= 6
Number of delayed SVs
wt ko dko

Fig. S6. Reduced spontaneous and strongly enhanced delayed quantal release is also observed in Cplx-deficient inhibitory small-bouton synapses of the brainstem and cerebellum.
Similar experiments as those illustrated in Fig. 3 performed in glycinergic MNTB→LSO synapses (**A-F**) and GABAergic interneuron of the cerebellar MLI→PC synapses (**G-L**).
A, Recording paradigm: MNTB PNs axons contacting neurons of the ipsilateral LSO were electrically stimulated in brainstem slices prepared from P15–19 mice. Pharmacologically isolated (2 μM NBQX) glycinergic IPSCs were recorded in voltage-clamped LSO PNs.
B, Sample traces of glycinergic AP-evoked IPSC trains recorded in a wt (*black*) and a ko (*red*) synapse.
C, Delayed quantal release following conditional stimulation (100 Hz, 15 APs) is prominent in Cplx1 ko (*red*) and Cplx1,Syt7 dko (*light red*) synapses, but nearly absent from synapses of wt littermate mice (*black*). Five consecutive sweeps are shown superimposed.
D, Average timecourse of $\xi(t)$ (AP-evoked IPSCs blanked for clarity) in wt (*black*) and ko (*red*) synapses. Solid lines and shaded areas represent mean and ± SEM, respectively.
E, Summary data for mean rates (*left*) and mean amplitudes (*right*) of sIPSC recorded in MNTB→LSO synapses of wt (*black*) and ko (*red*) mice. Lack of Cplx (*red*) or combined genetic ablation of Cplx1 and Syt7 (*light red*) strongly attenuates sIPSC frequency but does not affect sIPSC size.
F, Summary data for peak $\xi(t)$ (*left*) and total number of SVs (*right*) contributing to delayed quantal release analyzed over a time period of 1 s starting 10 ms after the last stimulus in the train. Lack of Cplx (*red*) or combined genetic ablation of Cplx1 and Syt7 (*light red*) strongly enhances peak $\xi(t)$ and total number of asynchronously released quanta.
G, Recording paradigm: Paired recordings were obtained from presynaptic MLIs and postsynaptic PCs which were maintained under current-clamp and voltage-clamp, respectively, in cerebellar slices prepared from P20–28 mice. APs were triggered in MLIs by injection of brief depolarizing current pulses (400 pA, 4 ms). Pharmacologically isolated (2 μM NBQX) GABAergic IPSCs were recorded in PCs.
H, Sample traces of GABAergic AP-evoked IPSCs trains recorded in a wt (*black*) and a ko (*red*) synapse.
I, Delayed quantal release following conditional stimulation (200 Hz, 25 APs) is prominent in Cplx1 ko (*red*) and Cplx1,Syt7 dko (*light red*) MLI→PC synapses, but less pronounced in synapses of wt littermate mice (*black*). Five consecutive sweeps are shown superimposed.
J, Average timecourse of $\xi(t)$ (AP-evoked IPSCs blanked for clarity) in wt (*black*) and ko (*red*) synapses. Solid lines and shaded areas represent mean and ± SEM, respectively.
K, Summary data for mean rates (*left*) and mean amplitudes (*right*) of spontaneously occurring IPSCs (sIPSC) recorded in MLI→PC synapses of wt (*black*) and ko (*red*) mice. Lack of Cplx (*red*) or combined genetic ablation of Cplx1 and Syt7 (*light red*) strongly attenuates sIPSC frequency but does not affect sIPSC size.
L, Summary data for peak $\xi(t)$ (*left*) and total number of SVs (*right*) contributing to delayed quantal release analyzed over a time period of 1 s starting 10 ms after the last stimulus in the train. Lack of Cplx (*red*) or combined genetic ablation of Cplx1 and Syt7 (*light red*) strongly enhances peak $\xi(t)$ and total number of asynchronously released quanta.
AP-evoked synchronous IPSCs are blanked in **C**,**D** and **I**,**J** for clarity.

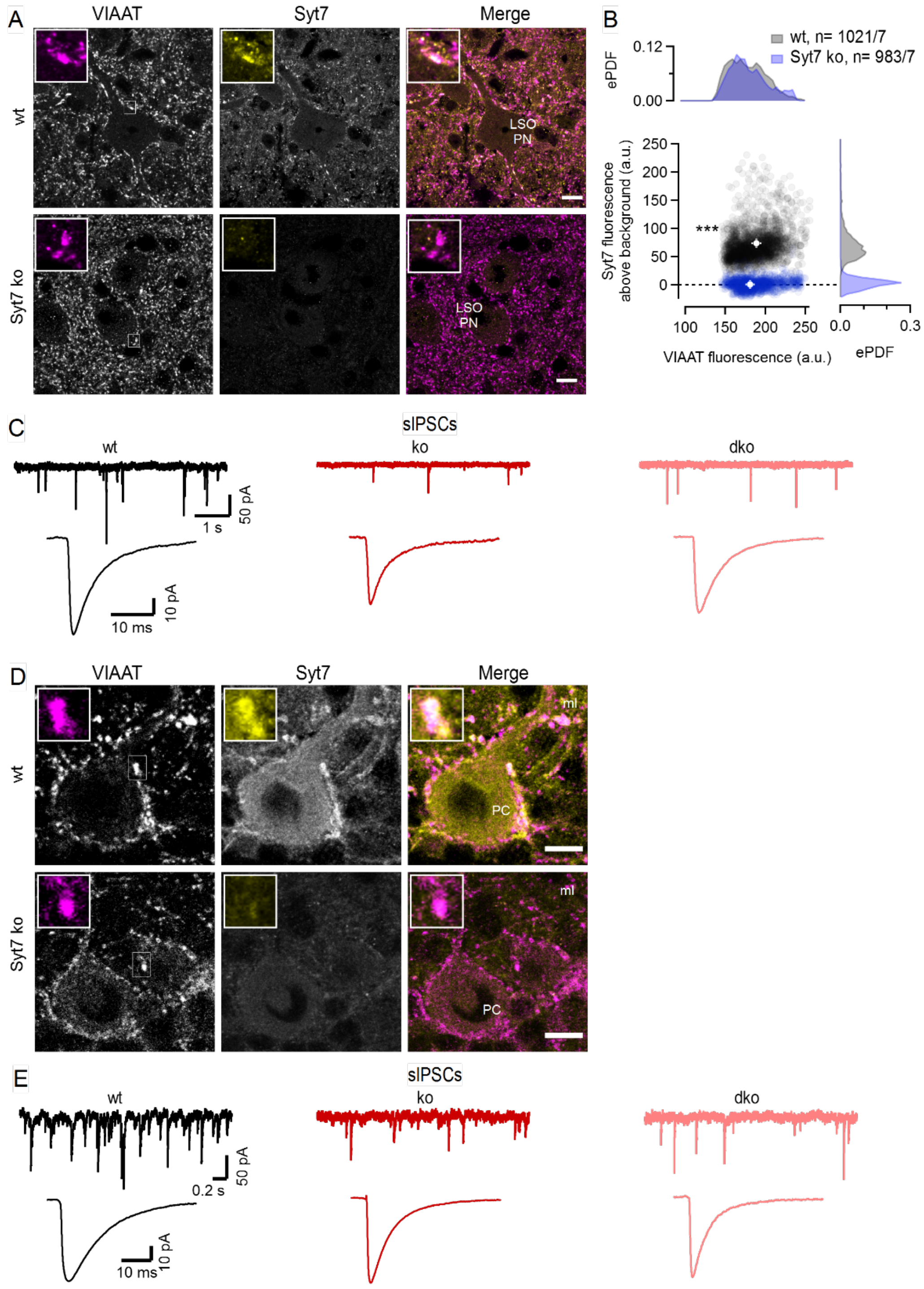

Fig. S7. Synaptotagmin 7 expression in glycinergic boutons contacting LSO PNs and in GABAergic boutons contacting cerebellar Purkinje cells and analysis of spontaneously occurring glycinergic and GABAergic IPSCs.

A, Syt7 expression in putative glycinergic MNTB→LSO synapses. Maximum-intensity projections were acquired from confocal image stacks of LSO PN somata decorated by putative presynaptic boutons immunolabeled for the presynaptic marker protein VIAAT (*left column*, magenta in the

right column) in sections obtained from a wt (*top row*) and a Syt7 ko (*bottom row*) mouse. Sections were co-stained with an anti-Syt7 antibody (*middle column*, yellow in the *right column*) to demonstrate presence and absence of Syt7 expression in inhibitory MNTB→LSO synapses of wt and Syt7 ko mice, respectively. Insets show VIAAT$^+$ putative glycinergic boutons at higher magnification. Scale bar, 10 µm.
B, Scatter plot of anti-Syt7 versus anti-VIAAT fluorescence intensity levels in VIAAT-positive (VIAAT$^+$) puncta surrounding LSO PNs in wt (*gray*) and Syt7 ko (*blue*) mice. Syt7 fluorescence levels were background-corrected by subtracting the average fluorescence measured of nonspecific regions outside of the tissue. Empirical probability density functions (ePDFs, bin width 5 a.u.) for anti-VIAAT fluorescence intensity and anti- Syt7 fluorescence intensity are shown above and to the right of the scatter plot, respectively.
C, Sample traces (*top*) and average waveforms (*bottom*) of spontaneously occurring glycinergic IPSCs (sIPSCs) recorded in LSO PNs of wt (*black*), Cplx1 ko (*red*), and Cplx1,Syt7 dko (*light red*) mice.
D, Syt7 expression in putative GABAergic MLI→PC synapses. Maximum-intensity projections were acquired from confocal image stacks of PC somata decorated by presynaptic boutons immunolabeled for the presynaptic marker protein VIAAT (*left column*, magenta in the *right column*) in sections obtained from a P22 wt (*top row*) and a P22 Syt7 ko (*bottom row*) mouse. Sections were co-stained with an anti-Syt7 antibody to demonstrate presence and absence of Syt7 expression in GABAergic MLI→PC synapses of wt and Syt7 ko mice, respectively. Insets show VIAAT$^+$ putative GABAergic boutons at higher magnification. Scale bar, 50 µm.
E, Sample traces (*top*) and average waveforms (*bottom*) of spontaneously occurring GABAergic IPSCs (sIPSCs) recorded in PCs in cerebellar slices obtained from of wt (*black*), Cplx1 ko (*red*), and Cplx1,Syt7 dko (*light red*) mice.

Table S1. Model parameter descriptions and parameter values for the sequential two-step priming and fusion scheme for wt and Cplx1 ko calyx synapses. (Parameter values which differ in Cplx ko in comparison to wt synapses are emphasized in red.)

Model parameter description	Symbol	Cplx1 wt	Cplx1 ko
Fast decay τ of the AP-induced 'effective' Ca^{2+} transient	τ_f	60 ms	60 ms
Slow decay τ of the AP-induced 'effective' Ca^{2+} transient	τ_s	260 ms	260 ms
Fraction of the slow decay component of the AP-induced 'effective' Ca^{2+} transient	f_s	0.12	0.12
Basal $[Ca^{2+}]_i$ at rest	$[Ca^{2+}]_{rest}$	50 nM	50 nM
$[Ca^{2+}]_i$ increment per AP-induced 'effective' Ca^{2+} transient	$\Delta[Ca^{2+}]$	110 nM	110 nM
Initial fusion probability	$p_{fusion,1}$	0.48	0.2
Total number of release sites	N_{tot}	2150	1900
Rate constant of the ES→LS transition at rest	$k_{1,rest}$	0.403 s^{-1}	0.403 s^{-1}
Ca^{2+} dependence of the ES→LS transition	σ_1	7.16 μM^{-1}	7.16 μM^{-1}
Rate constant of the ES←LS transition	b_1	0.185 s^{-1}	0.185 s^{-1}
Rate constant of the LS→TS transition at rest	$k_{2,rest}$	0.18 s^{-1}	0.18 s^{-1}
Ca^{2+} dependence of the LS→TS transition	σ_2	9.67 μM^{-1}	6.80 μM^{-1}
Rate constant of the LS←TS transition	b_2	0.28 s^{-1}	0.28 s^{-1}
Decay time constant of the TSL state, inverse of the rate constant of the LS←TSL transition	$\tau_{TSL} = 1 / b_3$	60 ms	60 ms
Fraction of LS SVs transferred to TSL SVs by an AP	κ	0.12	0.12
Rate constant for the ERS→ES transition	b_4	2.3 s^{-1}	2.3 s^{-1}
Exponent of power law of facilitation variable y		4.5	4.5
Increment of the facilitation variable y	y_{inc}	0.39	0.39
Decrement of the inactivation variable z	z_{dec}	0.4	0.4
Maximum value of the facilitation variable y	y_{max}	1.25	1.3
Minimum value of the inactivation variable z	z_{min}	0.75	0.75
Decay time constant of the facilitation variable y	τ_y	12 ms	12 ms
Decay time constant of inactivation variable z	τ_z	3 s	3 s
Scaling factor for the rate constant of the ES→FS transition at rest ($k_{f,rest} = s_k \cdot k_{1,rest}$)	s_k	0.0325	0.065
Scaling factor for the rate constant of the ES←FS transition at rest ($b_{f,rest} = s_b \cdot b_{1,rest}$)	s_b	14.0	7.0
Scaling factor for the Ca^{2+} dependence of the ES→LS transition ($\sigma_f = s_\sigma \cdot \sigma_1$)	s_σ	0.01	1.0

SI References

1. K. Reim *et al.*, Complexins regulate a late step in Ca^{2+}-dependent neurotransmitter release. *Cell* **104**, 71-81 (2001).
2. A. Maximov *et al.*, Genetic analysis of synaptotagmin-7 function in synaptic vesicle exocytosis. *Proc Natl Acad Sci U S A* **105**, 3986-3991 (2008).
3. S. Chang *et al.*, Complexin stabilizes newly primed synaptic vesicles and prevents their premature fusion at the mouse calyx of held synapse. *J Neurosci* **35**, 8272-8290 (2015).
4. C. Chen, R. Satterfield, S. M. Young, P. Jonas, Triple Function of Synaptotagmin 7 Ensures Efficiency of High-Frequency Transmission at Central GABAergic Synapses. *Cell Rep* **21**, 2082-2089 (2017).
5. H. Taschenberger, R. M. Leao, K. C. Rowland, G. A. Spirou, H. von Gersdorff, Optimizing synaptic architecture and efficiency for high-frequency transmission. *Neuron* **36**, 1127-1143 (2002).
6. H. Taschenberger, V. Scheuss, E. Neher, Release kinetics, quantal parameters and their modulation during short-term depression at a developing synapse in the rat CNS. *J Physiol* **568**, 513-537 (2005).
7. A. Y. Wong, B. P. Graham, B. Billups, I. D. Forsythe, Distinguishing between presynaptic and postsynaptic mechanisms of short-term depression during action potential trains. *J Neurosci* **23**, 4868-4877 (2003).
8. J. D. Clements, J. M. Bekkers, Detection of spontaneous synaptic events with an optimally scaled template. *Biophys J* **73**, 220-229 (1997).
9. E. Neher, H. Taschenberger, Non-negative Matrix Factorization as a Tool to Distinguish Between Synaptic Vesicles in Different Functional States. *Neuroscience* **458**, 182-202 (2021).
10. K. H. Lin, H. Taschenberger, E. Neher, A sequential two-step priming scheme reproduces diversity in synaptic strength and short-term plasticity. *Proc Natl Acad Sci U S A* **119**, e2207987119 (2022).
11. T. Sakaba, E. Neher, Quantitative relationship between transmitter release and calcium current at the calyx of Held synapse. *J Neurosci* **21**, 462-476 (2001).
12. T. Sakaba, E. Neher, Calmodulin mediates rapid recruitment of fast-releasing synaptic vesicles at a calyx-type synapse. *Neuron* **32**, 1119-1131 (2001).
13. N. Lipstein *et al.*, Munc13-1 is a Ca^{2+}-phospholipid-dependent vesicle priming hub that shapes synaptic short-term plasticity and enables sustained neurotransmission. *Neuron* **109**, 3980-4000 e3987 (2021).
14. E. Neher, Merits and Limitations of Vesicle Pool Models in View of Heterogeneous Populations of Synaptic Vesicles. *Neuron* **87**, 1131-1142 (2015).
15. X. Lou, V. Scheuss, R. Schneggenburger, Allosteric modulation of the presynaptic Ca^{2+} sensor for vesicle fusion. *Nature* **435**, 497-501 (2005).
16. O. Kochubey, R. Schneggenburger, Synaptotagmin increases the dynamic range of synapses by driving Ca^{2+}-evoked release and by clamping a near-linear remaining Ca^{2+} sensor. *Neuron* **69**, 736-748 (2011).
17. L. Y. Wang, E. Neher, H. Taschenberger, Synaptic vesicles in mature calyx of Held synapses sense higher nanodomain calcium concentrations during action potential-evoked glutamate release. *J Neurosci* **28**, 14450-14458 (2008).
18. M. Xue *et al.*, Complexins facilitate neurotransmitter release at excitatory and inhibitory synapses in mammalian central nervous system. *Proc Natl Acad Sci U S A* **105**, 7875-7880 (2008).

19. A. C. Davison, D. V. Hinkley, E. Schlechtman, Efficient Bootstrap Simulation. *Biometrika* **73**, 555-556 (1986).
20. J. R. Gleason, Algorithms for Balanced Bootstrap Simulations. *The American Statistician* **42**, 263-266 (1988).
